# Synthesis of ferrocenyl-substituted 1,3-dithiolanes via [3 + 2]-cycloadditions of ferrocenyl hetaryl thioketones with thiocarbonyl *S*-methanides

**DOI:** 10.3762/bjoc.12.136

**Published:** 2016-07-08

**Authors:** Grzegorz Mlostoń, Róża Hamera-Fałdyga, Anthony Linden, Heinz Heimgartner

**Affiliations:** 1 Department of Organic and Applied Chemistry, University of Łódź, Tamka 12, PL 91-403 Łódź, Poland; 2 Department of Chemistry, University of Zürich, Winterthurerstrasse 190, CH-8057 Zürich, Switzerland

**Keywords:** [3 + 2]-cycloadditions, 1,3-dithiolanes, ferrocene, thiocarbonyl *S*-methanides, thioketones

## Abstract

Ferrocenyl hetaryl thioketones react smoothly with in situ generated thiocarbonyl *S*-methanides to give 1,3-dithiolanes. In the case of aromatic *S*-methanides, the sterically more crowded 4,4,5,5-tetrasubstituted 1,3-dithiolanes (2-CH_2_ isomers) were formed as sole products. The reactions with cycloaliphatic *S*-methanides led to mixtures of 2-CH_2_ and 5-CH_2_ isomers with the major component being the sterically more crowded 2-CH_2_ isomers. The preferred formation of the latter products is explained by the assumption that the formal [3 + 2]-cycloadducts were formed via a stepwise reaction mechanism with a stabilized 1,5-diradical as a key intermediate. The complete change of the reaction mechanism toward the concerted [3 + 2]-cycloaddition was observed in the reaction of a sterically crowded cycloaliphatic thiocarbonyl ylide with ferrocenyl methyl thioketone.

## Introduction

In a recent publication, a straightforward method for the synthesis of hitherto little known ferrocenyl aryl/hetaryl thioketones of type **1** via a standard procedure by treatment of the corresponding ferrocenyl ketones with Lawesson reagent was described [1]. The latter substrates were prepared efficiently via acylation of ferrocene with in situ generated mixed anhydrides containing a trifluoroacetyl unit or, alternatively, by ferrocenylation of furan, thiophene or selenophene with mixed trifluoroacetyl anhydride. The obtained ferrocenyl thioketones are remarkably stable in comparison with their aromatic analogues. Thus, the convenient access to thioketones **1** permitted their exploration for the synthesis of diverse sulfur heterocycles based on the reactivity of compounds containing a C=S group, known as ‘superdipolarophiles’ [2] or ‘superdienophiles’ [3].

The reactive thiocarbonyl *S*-methanides of type **2** can conveniently be generated by thermal elimination of N_2_ from 1,3,4-thiadiazoles **3** at ca. −45 °C in the case of 2,2-diaryl derivatives **3a**,**b** or upon gentle heating of cycloaliphatic precursors **3c**,**d** in THF solution to ca. +45 °C [4–5]. The [3 + 2]-cycloaddition of dipoles **2** with thioketones is well known as an excellent method for the construction of tetrasubstituted 1,3-dithiolanes [5]. A remarkable feature of these reactions is the regioselectivity. Whereas aromatic *S*-methanides react to give the sterically crowded 4,4,5,5-tetrasubstituted 1,3-dithiolanes [6–7], cycloaliphatic *S*-methanides tend to form mixtures of both regioisomeric cycloadducts with the major component being the sterically more crowded isomer [7–8]. In a recent study, we proposed a diradical mechanism for the [3 + 2]-cycloadditions of thiocarbonyl *S*-methanides with aromatic and heteroaromatic thioketones [7,9]. Furthermore, the analogous reaction mechanism was postulated to explain the results obtained with thiocarbonyl *S*-ethanides, *S*-isopropanides, and *S*-(trimethylsilyl)methanides [10].

Ferrocenyl thioketones have never been exploited in reactions with thiocarbonyl *S*-methanides. On the other hand, ferrocenyl-substituted 1,3-dithiolanes can be of interest for materials chemistry and for electrochemical studies [11]. The goal of the present study was to examine reactions of selected ferrocenyl hetaryl thioketones **1** with aromatic and cycloaliphatic thiocarbonyl *S*-methanides **2**. Preliminary results of this study have been presented at the IRIS-14^th^ conference as a poster communication (see [12]).

## Results and Discussion

**Scheme 1:**
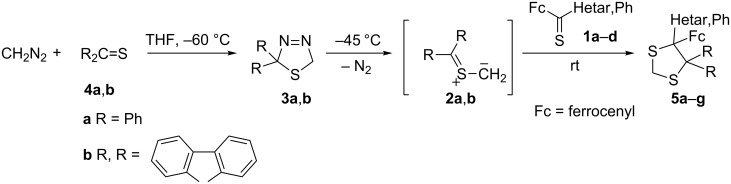
Reactions of aromatic thiocarbonyl *S*-methanides **2a**,**b** with ferrocenyl thioketones **1** (Table 1).

The precursors of aromatic *S*-methanides **2a**,**b**, i.e., 1,3,4-thiadiazolines **3a**,**b**, were prepared from thiobenzophenone (**4a**) and thiofluorenone (**4b**), respectively, and diazomethane at –60 °C in THF solution [6] (Scheme 1). After addition of an equimolar amount of a ferrocenyl hetaryl thioketone **1**, the reaction mixture was slowly warmed to room temperature. The crude mixture was examined by ^1^H NMR spectroscopy, and in all cases only one single product **5** was detected (Table 1).

**Table 1:** Ferrocenyl-substituted 1,3-dithiolanes **5** and **6**.

**5**/**6**	R, R	Hetar, Ph	Ratio of **5**/**6**^a^	Yield of **5** + **6** [%]^b^

**a**	Ph, Ph	furan-2-yl	100:0	49
**b**	Ph, Ph	thiophen-2-yl	100:0	57
**c**	Ph, Ph	selenophen-2-yl	100:0	62
**d**	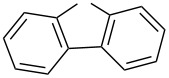	furan-2-yl	100:0	33
**e**	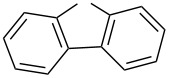	thiophen-2-yl	100:0	32
**f**	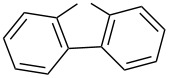	selenophen-2-yl	100:0	32
**g**	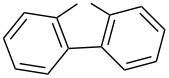	Ph	100:0	40
**h**	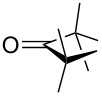	furan-2-yl	76:24	62^c^
**i**	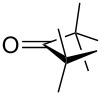	thiophen-2-yl	93:7	79^c^
**j**	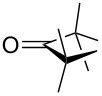	selenophen-2-yl	78:22	53^c^
**k**	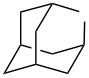	thiophen-2-yl	75:25	57^c^
**l**	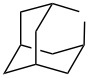	selenophen-2-yl	67:33	73^c^

^a^Determined by ^1^H NMR spectroscopy in the crude mixture. ^b^Yields of isolated product after chromatography and crystallization. ^c^Yields of isolated mixtures of products.

For example, 1,3-dithiolane **5a** showed a characteristic AB-system for the CH_2_ group and only one singlet for five equivalent CH groups of the unsubstituted cyclopentadiene unit of the ferrocenyl skeleton. On the other hand, the ^13^C NMR spectrum of the isolated sample **5a** revealed the signal of the CH_2_ group at 30.8 ppm, typical for 4,4,5,5-tetrasubstituted 1,3-dithiolanes (2-CH_2_ type [13]). Analogously, single 1,3-dithiolanes **5b**–**f** were obtained, and the ^13^C NMR spectra showed the CH_2_ absorption in the same region (27.3–33.0 ppm). Also in the case of 1,3-dithiolane **5g**, bearing a phenyl group, the absorption of the CH_2_ group was found at 31.2 ppm.

**Figure 1:**
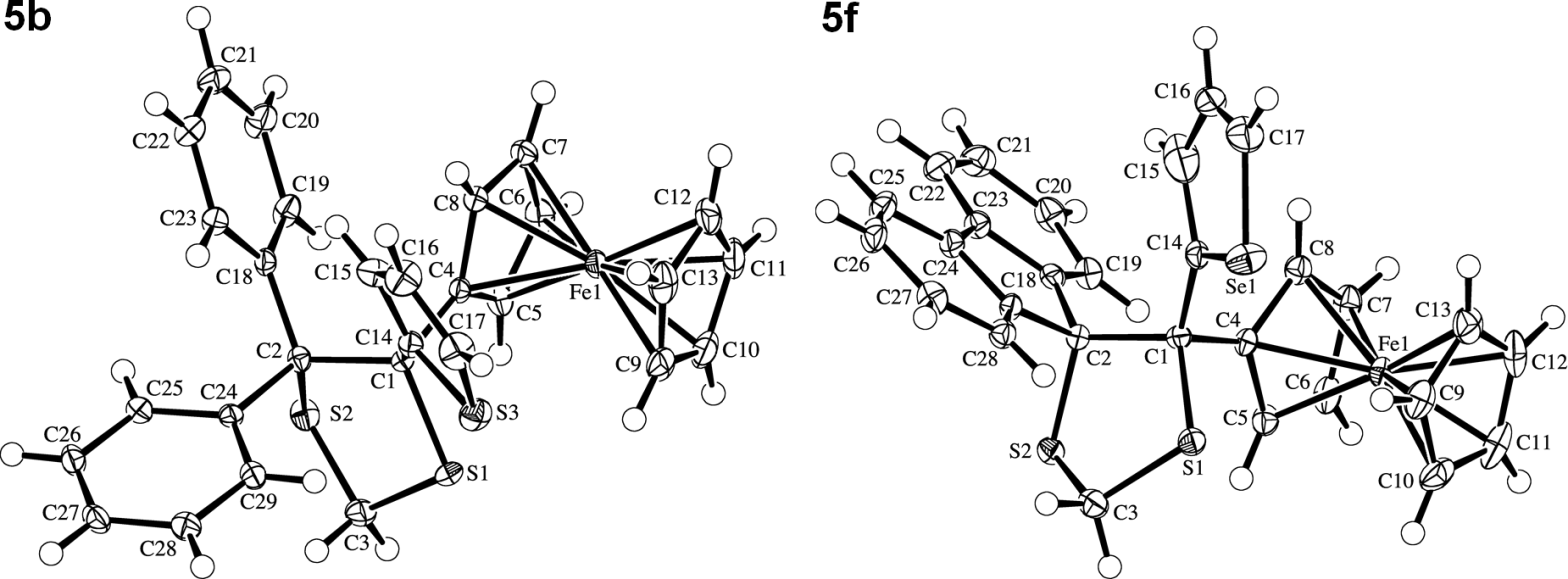
ORTEP Plot [16] of the molecular structures of the ferrocenyl-substituted 1,3-dithiolanes **5b** and **5f** (with 50% probability ellipsoids; arbitrary numbering of the atoms; only the major disorder conformation of the selenophene ring is shown).

The structures of the ferrocenyl-substituted 1,3-dithiolanes **5b**, **5e**, **5f**, and **5g** have been established unambiguously by X-ray crystallography (see Figure 1 for **5b** and **5f**). The space group of **5b** is non-centrosymmetric with a polar axis, but achiral. Refinement of the absolute structure parameter [14–15] yielded a value of 0.13(1), which indicates that the crystal is a partial inversion twin with a major twin fraction of 0.87(1). In the cases of **5e** and **5f**, the thiophene and selenophene rings are disordered over two orientations related by a 180° flip of the ring about its bonding axis, with the major conformation present in 88% and 91% of the molecules, respectively.

**Scheme 2:**
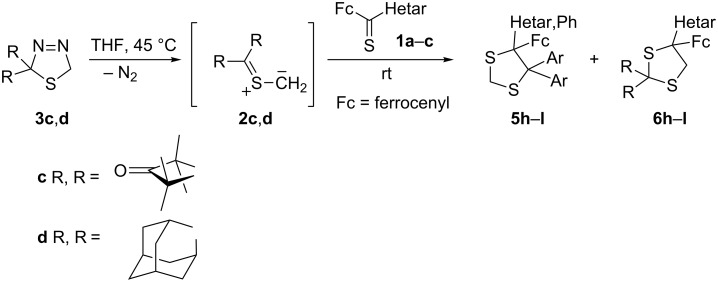
Reactions of cycloaliphatic thiocarbonyl *S*-methanides with ferrocenyl hetaryl thioketones **1** (Table 1).

In similar experiments, 2,2,4,4-tetramethyl-3-thioxocyclobutanone *S*-methanide (**2c**) and adamantanethione *S*-methanide (**2d**), respectively, were generated in situ at 45 °C from the corresponding precursors **3c**,**d**. In the presence of an equimolar amount of **1a**–**c**, they were trapped to give mixtures of two regioisomeric 1,3-dithiolanes **5** and **6** (Scheme 2). In all cases, the ^1^H NMR analysis of the crude mixtures indicated that the major products obtained in these reactions were the sterically more crowded 1,3-dithiolanes **5**. After separation of the products, the ^13^C NMR spectra of the major isomers showed the signal for H_2_C(2) in the typical region (25.7–28.9 ppm). The minor products were identified on the basis of the ^13^C NMR spectra of the crude mixtures. In this series, the characteristic signals of H_2_C(5) appeared at 49.3–53.9 ppm, in agreement with literature data [13].

**Scheme 3:**
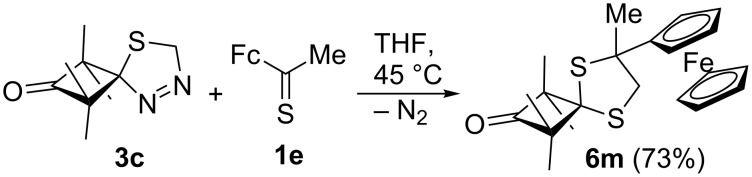
Reaction of **3c** with ferrocenyl methyl thioketone **1e**.

In an extension of the study with ferrocenyl hetaryl thioketones **1**, an experiment with ferrocenyl methyl thioketone (**1e**) and thiocabonyl *S*-methanide **2c** was performed under typical conditions (THF, 45 °C). Unexpectedly, in this case, the ^1^H NMR spectrum of the crude product revealed the presence of only one 1,3-dithiolane, i.e., only one set of five methyl signals at 1.36, 1.38, 1.41, 1.42, and 1.97 ppm was present. The AB-system of the CH_2_ group appeared at 2.97 and 3.25 ppm with *J* = 12.0 Hz. After chromatographic purification, the ^13^C NMR spectrum confirmed the 5-CH_2_-type of the 1,3-dithiolane **6m**, as the corresponding CH_2_ absorption was found at 52.5 ppm (Scheme 3).

**Scheme 4:**
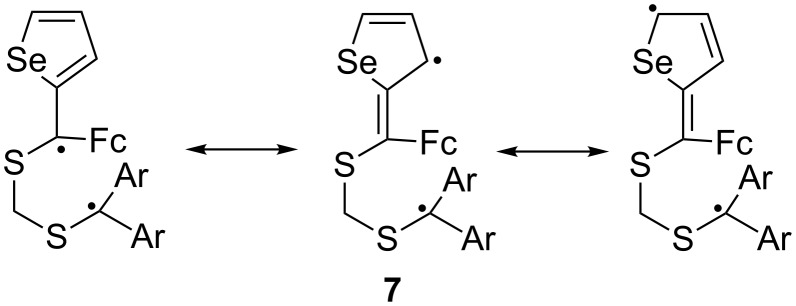
Mesomeric stabilization of the 1,5-diradical intermediate in the reaction of aromatic thiocarbonyl *S*-methanides with ferrocenyl hetaryl thioketones.

Based on our earlier interpretation of the reaction mechanism leading to 1,3-dithiolanes via [3 + 2]-cycloaddition of thiocarbonyl *S*-methanides with aryl and hetaryl thioketones, we propose that the reactions with ferrocenyl thioketones **1** occur predominantly via an intermediate 1,5-diradical. The formation of the sterically more crowded 1,3-dithiolanes **5a**–**g** confirms that the stabilized 1,5-diradicals of type **7** (Scheme 4) are key-intermediates in the reaction. On the other hand, the formation of 1,3-dithiolanes of type **5** in reactions with cycloaliphatic thiocarbonyl *S*-methanides competes with the concerted [3 + 2]-cycloaddition leading to the sterically less crowded 1,3-dithiolanes **6** (Scheme 2). These results demonstrate that the ferrocenyl moiety shows a comparable effect to that found for aryl and hetaryl groups. This interpretation of the reaction mechanism got additional support from the reaction of **1e** with **3c**. Apparently, the replacement of a radical-stabilizing aryl or hetaryl group by a methyl substituent results in the preferred concerted [3 + 2]-cycloaddition. It is well known that this mechanism, controlled by frontier-orbital (FMO) interactions, is strongly influenced by steric effects in the case of C=S dipolarophiles [8,17].

## Conclusion

The presented study indicated that ferrocenyl thioketones show an enhanced stability in comparison with aromatic thioketones. Therefore, they are considered as promising substrates for the synthesis of more complex sulfur-containing compounds. The reactions with electron-rich thiocarbonyl *S*-methanides occur smoothly to give 1,3-dithiolanes as products of formal [3 + 2]-cycloadditions. The high reactivity of ferrocenyl thioketones is demonstrated by the fact that no products of competitive reactions, such as dimerization of the intermediate 1,3-dipole or 1,3-dipolar electrocyclizations, were observed. Based on these observations one can expect that ferrocenyl thioketones may also be used as prone dipolarophiles in reactions with other dipoles. Similarly, they are expected to display a high dienophilicity.

The results obtained with thiocarbonyl *S*-methanides, i.e., the preferred formation of the sterically more crowded 4,4,5,5-tetrasubstituted 1,3-dithiolanes, supports the proposed stepwise radical reaction mechanism via a stabilized 1,5-diradical as an intermediate. Finally, in the case of ferrocenyl methyl thioketone used as a dipolarophile, the concerted [3 + 2]-cycloaddition dominated due to the reduced stabilizing effect of the methyl group compared with that of a hetaryl substituent.

## Experimental

**General information:** All solvents were dried over appropriate drying agents and distilled before use. Melting points were determined in a capillary using a Stewart^®^ SMP30 apparatus. The IR spectra (KBr pellets) were recorded on a Nexus FTIR spectrometer. The ^1^H and ^13^C NMR spectra were measured on a Bruker Avance III (600 and 150 MHz, respectively) instrument in CDCl_3_, using the solvent signal as reference. ESIMS were recorded on a Varian 500-MS IT Mass Spectrometer. The elemental analyses were performed on a Vario Micro Cube apparatus. Flash chromatography was carried out using Silica gel 60 (Sigma-Aldrich, 230–400 mesh). The notation Fc in this study represents ferrocenyl. Applied reagents such as ferrocenyl-substituted thioketones (**1**) [1], thiobenzophenone (**4a**) [18], and thiofluorenone (**4b**) [19] were prepared using known procedures. 1,1,3,3-Tetramethyl-8-thia-5,6-diazaspiro[3.4]oct-5-en-2-one (**3c**) [20] and spiro[1,3,4-thiadiazole-2(5*H*),2’-tricyclo[3.3.1.1^3,7^]decane] (**3d**) [21] were obtained by known methods according to the literature protocols. Diazomethane was prepared in a 5 mmol scale either from *N*-nitroso-*N*-methylurea or from *N*-nitrosotoluene-4-sulfonylmethylamide by treatment with aqueous KOH solution in a two-phase system with diethyl ether at room temperature. After separation of the ethereal phase it was dried over KOH pellets and used for further experiments with no distillation. Other reagents used in the present study were commercially available.

**Preparation of diaryl-substituted 1,3-dithiolanes (5a**–**g)** – **General procedure:** A solution of thioketone **4a** or **4b** (1 mmol) in THF (3 mL) was cooled to –75 °C (acetone/dry ice). Then, the mixture was treated with small portions of ethereal diazomethane solution, until the disappearance of the characteristic color of the thioketone (**4a**,**b**). Excess of diazomethane was removed under reduced pressure at −75 °C. Then, a solution of ferrocenyl hetaryl thioketone **1** (1 mmol) in THF (4 mL) was added to the mixture, which was allowed to warm slowly to rt. Next, the solvent was evaporated, and the crude products were purified by crystallization (hexane/CH_2_Cl_2_).

**Preparation of cycloaliphatic-substituted 1,3-dithiolanes (5h**–**l**, **6h**–**m)** – **General procedure:** To a solution of the corresponding ferrocenyl-substituted thioketone **1** (1 mmol) in THF (4 mL), 1,3,4-thiadiazoline **3c** or **3d** (1 mmol) was added. The mixture was heated at 45 °C until the evolution of N_2_ ceased. Then, the solvent was evaporated and the crude products were purified by CC (SiO_2_, hexane/ethyl acetate 95:5). The major product was isolated using diethyl ether (precipitation).

**4-Ferrocenyl-5,5-diphenyl-4-(selenophen-2-yl)-1,3-dithiolane** (**5c**): Yield: 354 mg (62%). Orange crystals; mp >162 °C (decomposition); IR (KBr) ν: 3094 (m), 3030 (w), 2967 (w), 2911 (m), 1595 (w), 1577 (w), 1488 (m), 1440 (s), 1409 (m), 1391 (m), 1233 (s), 1216 (m), 1188 (m), 1105 (s), 1084 (m), 1061 (m), 1044 (m), 1030 (s), 1001 (m), 950 (m), 816 (s), 745 (s), 717 (vs), 697 (vs), 677 (vs), 641 (m), 603 (m), 502 (vs), 490 (s) cm^–1^; ^1^H NMR (600 MHz, CDCl_3_) δ 7.91 (d, *J*_H,H_ = 6.0 Hz, 1H, H_arom._), 7.55–7.54 (m, 3H, H_arom._), 7.23–7.21 (m, 2H, H_arom._), 7.18–7.07 (m, 7H, H_arom._), 4.12 (bs, 1H, H-Fc), 4.09 (bs, 6H, H-Fc), 4.04 (bs, 1H, H-Fc), 3.94 (bs, 1H, H-Fc), 3.91, 3.87 (AB system, *J*_H,H_ = 9.0 Hz, 2H, CH_2_) ppm; ^13^C NMR (150 MHz, CDCl_3_) δ 154.7, 143.1 (3 C_arom._), 132.2, 131.0, 130.7, 130.0, 128.1, 127.1, 126.8, 126.5, 126.1 (13 CH_arom._), 90.5, 78.8, 74.8 (C-Fc, 2 C_q_), 72.2, 72.1 (2 CH-Fc), 69.8 (5 CH-Fc), 67.7, 67.1 (2 CH-Fc), 30.8 (CH_2_) ppm; ESIMS *m/z* (%): 572 (100, [M + H]^+^), 571 (60, [M^+^]); anal. calcd for C_29_H_24_FeS_2_Se (571.44): C, 60.95; H, 4.23; S, 11.22; found: C, 61.04; H, 4.33; S, 11.01.

**5-Ferrocenyl-5-phenylspiro[1,3-dithiolane-4,9’-[9*H*]fluorene]** (**5g**): Yield: 206 mg (40%). Orange crystals; mp >185 °C (decomposition); IR (KBr) ν: 3057 (m), 2918 (m), 1636 (m), 1597 (m), 1492 (m), 1474 (m), 1445 (vs), 1414 (m), 1390 (m), 1286 (m), 1225 (m), 1157 (m), 1108 (m), 1051 (m), 1035 (m), 1000 (m), 863 (m), 816 (s), 746 (vs), 730 (vs), 710 (s), 698 (m), 521 (m), 503 (m), 484 (m) cm^–1^; ^1^H NMR (600 MHz, CDCl_3_) δ 7.67 (dd, *J*_H,H_ = 7.8, 10.8 Hz, 2H, H_arom._), 7.59 (d, *J*_H,H_ = 7.8 Hz, 1H, H_arom._), 7.36 (t, *J*_H,H_= 7.8 Hz, 1H, H_arom._), 7.15 (t, *J*_H,H_ = 7.8 Hz, 1H, H_arom._), 7.02–6.98 (m, 5H, H_arom._), 6.24 (d, *J*_H,H_= 4.2 Hz, 1H, H_arom._), 5.06 (bs, 1H, H-Fc), 4.50–4.49 (m, 1H, H-Fc), 4.28, 4.20 (AB system, *J*_H,H_= 9.6 Hz, 2H, CH_2_), 4.08–4.07 (m, 1H, H-Fc), 4.04 (s, 5H, H-Fc), 3.53 (bs, 1H, H-Fc) ppm; ^13^C NMR (150 MHz, CDCl_3_) δ 149.5, 142.3, 141.0, 140.9, 138.1 (5 C_arom._), 128.7, 127.9, 127.5, 127.3, 126.6, 126.5, 124.8, 119.8, 119.7 (13 CH_arom._), 98.2, 74.8, 74.5 (C-Fc, 2 C_q_), 73.5, 71.6 (2 CH-Fc), 69.1 (for 5 CH-Fc), 69.0, 67.6 (2 CH-Fc), 31.2 (CH_2_) ppm; ESIMS *m/z* (%): 517 (43, [M + H]^+^), 516 (100, [M]^+^); anal. calcd for C_31_H_24_FeS_2_ (516.50): C, 72.09; H, 4.68; S, 12.42; found: C, 71.97; H, 4.70; S, 12.26.

**8-Ferrocenyl-1,1,3,3-tetramethyl-8-(thiophen-2-yl)-5,7-dithiaspiro[3.4]octan-2-one** (**5i**): Isolated as the major product. Yield: 381 mg (79%; crude product ratio 93:7). Orange solid; mp 190.2–192.0 °C; IR (KBr) ν: 3137 (w), 3112 (w), 3066 (m), 3042 (w), 3021 (m), 2965 (m), 2929 (s), 2866 (m), 1776 (vs, C=O), 1469 (m), 1455 (m), 1382 (m), 1362 (m), 1231 (m), 1165 (m), 1131 (m), 1106 (m), 1056 (m), 1022 (m), 1003 (m), 905 (m), 825 (s), 815 (s), 779 (m), 694 (vs), 486 (vs) cm^–1^; ^1^H NMR (600 MHz, CDCl_3_) δ 7.32 (dd, *J*_H,H_ = 3.6, 1.2 Hz, 1H, H_arom._), 7.29 (dd, *J*_H,H_ = 1.2, 5.4 Hz, 1H, H_arom._), 7.11 (dd, *J*_H,H_ = 3.6, 4.8 Hz, 1H, H_arom._), 4.91–4.90 (m, 1H, H-Fc), 4.54–4.53 (m, 1H, H-Fc), 4.25–4.24 (m, 1H, H-Fc), 4.15–4.14 (m, 1H, H-Fc), 3.94 (s, 5H, H-Fc), 3.78, 3.74 (AB system, *J*_H,H_ = 8.4 Hz, 2H, CH_2_), 1.72, 1.64, 1.19, 1.17 (4s, 12H, CH_3_) ppm; ^13^C NMR (150 MHz, CDCl_3_) δ 153.2 (C=O), 126.6, 123.3, 123.2 (3 CH_arom._), 72.2, 73.0 (2 CH-Fc), 70.1 (5 CH-Fc), 66.3, 68.6 (2 CH-Fc), 84.9, 76.5, 71.0, 70.7, 69.4, 62.7 (C_arom._, C-Fc, 4 C_q_), 26.1, 24.9, 24.1, 23.3 (4 CH_3_), 28.3 (CH_2_) ppm; ESIMS *m*/*z* (%): 583 (53, [M + H]^+^), 482 (100, [M]^+^); anal. calcd for C_24_H_26_FeOS_3_ (482.50): C, 59.74; H, 5.43; S, 19.94; found: C, 59.99; H, 5.40; S, 19.62.

**6-Ferrocenyl-1,1,3,3-tetramethyl-6-(selenophen-2-yl)-5,8-dithiaspiro[3.4]octan-2-one** (**6i**) (from the spectra of a mixture of **6i** with the major product **5i**): ^1^H NMR (600 MHz, CDCl_3_) δ 7.26 (dd, *J*_H,H_ = 5.4, 1.2 Hz, 1H, H_arom._), 7.14 (dd, *J*_H,H_ = 1.2, 3.6 Hz, 1H, H_arom._), 7.03 (dd, *J*_H,H_ = 3.6, 5.4 Hz, 1H, H_arom._), 4.37–4.36 (m, 1H, H-Fc), 4.23–4.22 (m, 1H, H-Fc), 4.22 (s, 5H, H-Fc), 4.14–4.13 (m, 1H, H-Fc), 4.02–4.01 (m, 1H, H-Fc), 3.63, 3.60 (AB system, *J*_H,H_ = 12.0 Hz, 2H, CH_2_), 1.48, 1.44, 1.41, 1.13 (s, 12H, CH_3_) ppm; ^13^C NMR (150 MHz, CDCl_3_) δ 150.7 (C=O), 126.4, 125.2, 124.3 (3 CH_arom._), 69.4, 68.3, 68.2, 68.0, 67.4 (9 CH-Fc), 92.8, 75.3, 69.0, 67.1, 66.2 (C_arom._, C-Fc, 4 C_q_), 53.3 (CH_2_), 25.1, 24.4, 22.8, 22.0 (4 CH_3_) ppm.

**5-Ferrocenyl-5-(selenophen-2-yl)spiro[1,3-dithiolane-4,2’-tricyclo[3.3.1.1^3,7^]decane]** (**5l**); **4-Ferrocenyl-4-(selenophen-2-yl)spiro[1,3-dithiolane-2,2’-tricyclo[3.3.1.1^3,7^]decane]** (**6l**): Isolated as a mixture of regioisomers. Yield: 393 mg (73%; crude product ratio 67:33). Yellow solid; IR (KBr) ν: 3085 (w), 3072 (w), 3012 (w), 2974 (w), 2901 (vs), 2854 (s), 1473 (w), 1442 (m), 1385 (w), 1347 (w), 1255 (w), 1233 (m), 1103 (m), 1036 (m), 998 (m), 970 (w), 929 (w), 824 (s), 808 (s), 755 (w), 720 (w), 701 (vs), 495 (s), 479 (s) cm^–1^; ^1^H NMR (600 MHz, CDCl_3_) δ 7.99 (d, *J*_H,H_ = 6.0 Hz, 1H, H_arom._), 7.90 (dd, *J*_H,H_ = 0.6, 5.4 Hz, 1H, H_arom._), 7.41 (dd, *J*_H,H_ = 6.0, 3.6 Hz, 1H, H_arom._), 7.24 (dd, *J*_H,H_ = 6.0, 3.6 Hz, 1H, H_arom._), 7.19 (dd, *J*_H,H_ = 1.2, 3.6 Hz, 1H, H_arom._), 7.14 (d, *J*_H,H_ = 3.6 Hz, 1H, H_arom._), 4.93–4.92 (m, 1H, H-Fc), 4.41 (bs, 1H, H-Fc), 4.30–4.29 (m, 1H, H-Fc), 4.24 (bs, 5H, H-Fc), 4.23–4.22 (m, 1H, H-Fc), 4.21–4.20 (m, 1H, H-Fc), 4.19–4.18 (m, 1H, H-Fc), 4.13–4.12 (m, 1H, H-Fc), 4.04 (s, 5H, H-Fc), 3.95, 3.64 (AB system, *J*_H,H_ = 8.4 Hz, 2H, CH_2_ (major)), 3.89, 3.58 (AB system, *J*_H,H_ = 12.6 Hz, 2H, CH_2_ (minor)), 2.96 (d, *J*_H,H_ = 12.6 Hz, 1H), 2.47–1.27 (m, 27H) ppm; ^13^C NMR (150 MHz, CDCl_3_) δ 161.3, 160.6 (2 C_arom._) 130.1, 129.9, 129.8, 129.3, 126.6, 125.1 (6 CH_arom._), 94.0, 87.7 (2 C-Fc), 77.9, 75.9, 74.7, 69.9 (4 C_q_), 72.5, 71.4, 70.0, 69.4, 69.0, 68.4, 67.8, 67.7, 67.3 (18 CH-Fc), 42.6, 41.8, 34.7, 34.1, 27.2, 27.1, 26.5, 26.4 (8 CH), 52.1, 40.4, 39.1, 37.7, 37.6, 37.4, 37.1, 35.9, 35.8, 33.6, 33.1, 26.0 (12 CH_2_) ppm; ESIMS *m/z* (%): 563 (60, [M + Na]^+^), 541 (40, [M + 2H]^+^), 540 (100, [M + H]^+^); anal. calcd for C_26_H_28_FeS_2_Se (539.44): C, 57.89; H, 5.23; S, 11.89; found: C, 57.96; H, 5.31; S, 11.92.

**6-Ferrocenyl-1,1,3,3,6-pentamethyl-5,8-dithiaspiro[3.4]octan-2-one** (**6m**): Yield: 302 mg (73%). Yellow crystals; mp 140.0–142.0 °C; IR (KBr) ν: 3094 (w), 3079 (w), 2968 (m), 2923 (m), 1777 (vs, C=O), 1752 (m), 1635 (m), 1458 (m), 1446 (m), 1378 (m), 1363 (m), 1277 (m), 1239 (m), 1169 (m), 1106 (m), 1055 (m), 1030 (m), 998 (m), 935 (m), 837 (m), 819 (m), 482 (m) cm^–1^; ^1^H NMR (600 MHz, CDCl_3_) δ 4.44 (bs, 1H, H-Fc), 4.23–4.21 (m, 7H, H-Fc), 4.17 (bs, 1H, H-Fc), 3.25, 2.97 (AB system, *J*_H,H_ = 12.0 Hz, 2H, CH_2_), 1.97, 1.42, 1.41, 1.38, 1.36 (5bs, 5H, CH_3_) ppm; ^13^C NMR (150 MHz, CDCl_3_) δ 220.6 (C=O), 93.1, 75.4 (C-Fc, 1 C_q_), 68.9 (5 CH-Fc), 68.2, 68.0, 67.3, 66.3 (4 CH-Fc), 67.0, 66.0, 63.9 (3 C_q_), 52.5 (CH_2_), 29.3, 25.0, 24.4, 22.8, 22.3 (5 CH_3_) ppm; ESIMS *m*/*z* (%): 416 (63, [M + 2H]^+^), 415 (100, [M + H]^+^); anal. calcd for C_21_H_26_FeOS_2_ (414.41): C, 60.86; H, 6.32; S, 15.48; found: C, 60.87; H, 6.38; S, 15.47.

## Supporting Information

CCDC-1469435–1469438 contain the supplementary crystallographic data for this paper. These data can be obtained free of charge from The Cambridge Crystallographic Data Centre via http://www.ccdc.cam.ac.uk/getstructures.

File 1 Experimental data for selected compounds **5** and **6**, details of the crystal structure determination, and the original ^1^H and ^13^C NMR spectra for all products.
