# Supplementary material for: Synthesis of ferrocenyl-substituted 1,3-dithiolanes via [3 + 2]-cycloadditions of ferrocenyl hetaryl thioketones with thiocarbonyl S-methanides
Source: Beilstein J Org Chem. 2016 Jul 8;12:1421–7. doi: 10.3762/bjoc.12.136 (PMC4979640; doi:10.3762/bjoc.12.136)
Supplement: File 1 — Experimental data for selected compounds 5 and 6, details of the crystal structure determination, and the original 1H and 13C NMR spectra for all products. [file Beilstein_J_Org_Chem-12-1421-s001.pdf]

Supporting Information

for

Synthesis of ferrocenyl-substituted 1,3-dithiolanes via [3 + 2]-cycloadditions of ferrocenyl hetaryl thioketones with thiocarbonyl *S*-methanides

Grzegorz Mlostoń[*1], Róża Hamera-Fałdyga[1], Anthony Linden[2] and Heinz Heimgartner[2]

Address: [1]Department of Organic and Applied Chemistry, University of Łódź, Tamka 12, PL 91-403 Łódź, Poland and [2]Department of Chemistry, University of Zürich, Winterthurerstrasse 190, CH-8057 Zürich, Switzerland

Email: Grzegorz Mlostoń - gmloston@uni.lodz.pl

*Corresponding author

Experimental data for selected compounds 5 and 6, details of the crystal structure determination, and the original ^{1}H and ^{13}C NMR spectra for all products

1. Experimental data for compounds 5, 6.

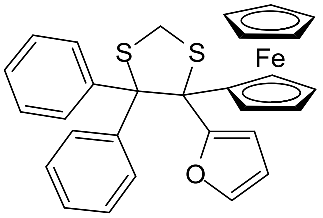

4-Ferrocenyl-4-(furan-2-yl)-5,5-diphenyl-1,3-dithiolane (**5a**): Yield: 249 mg (49%). Orange crystals; m.p.: ca. 190 °C (decomposition). IR (KBr): ν = 3110 (m), 3085 (m), 3034 (m), 2974 (m), 2863 (m), 1967 (w), 1958 (w), 1894 (w), 1818 (w), 1765 (w), 1654 (m), 1622 (m), 1578 (m), 1492 (vs), 1435 (s), 1413 (m), 1391 (m), 1217 (m), 1192 (m), 1147 (s), 1109 (m), 1052 (m), 1033 (m), 1017 (vs), 998 (m), 960 (m), 922 (m), 815 (vs), 751 (s), 732 (vs), 723 (vs), 698 (vs), 669 (m), 596 (m), 504 (vs), 489 (s) cm^{-1}. ^{1}H NMR (600 MHz, $CDCl_3$): δ = 7.47–7.49 (m, 2 $H_{arom.}$), 7.33 (bs, 1 $H_{arom.}$), 7.18–7.20 (m, 2 $H_{arom.}$), 7.08–7.15 (m, 4 $H_{arom.}$), 7.02–7.05 (m, 2 $H_{arom.}$), 6.60 (d, $J_{H,H}$ = 3.0 Hz,1 $H_{arom.}$), 6.40 (bs, 1 $H_{arom.}$), 4.66 (bs, 1 H-Fc), 4.13 (bs, 1 H-Fc), 3.97 (s, 5 H-Fc), 3.78 (bs, 1 H-Fc), 3.84, 3.62 (AB system, $J_{H,H}$ = 10.2 Hz, CH_2), 3.30 (bs, 1 H-Fc) ppm. ^{13}C NMR (150 MHz, $CDCl_3$): δ = 155.6, 144.0, 139.9 (3 $C_{arom.}$), 140.0, 131.8, 130.0, 127.0, 126.6, 126.1, 126.0, 110.4, 110.3 (13 $CH_{arom.}$), 94.3, 78.8, 70.2 (C-Fc, 2 C_q), 73.0, 71.0 (2 CH-Fc), 68.9 (for 5 CH-Fc), 67.7, 67.2 (2 CH-Fc), 30.8 (CH_2) ppm. MS (ESI): *m/z* (%): 509 (35, $[M+H]^+$), 508 (100, $[M]^+$). Anal. calcd. for $C_{29}H_{24}FeOS_2$ (508.48): C 68.50, H 4.76, S 12.61; found: C 68.51, H 4.78, S 12.61.

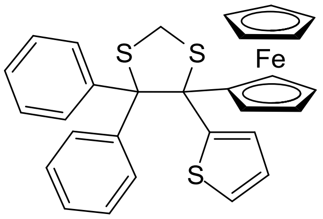

4-Ferrocenyl-5,5-diphenyl-4-(thiophen-2-yl)-1,3-dithiolane (**5b**): Yield: 299 mg (57%). Yellow crystals; m.p.: ca. 195 °C (decomposition). IR (KBr): ν = 3095 (w), 3071 (w), 3061 (w), 3031 (w), 2920 (w), 1636 (w), 1489 (m), 1442 (m), 1408 (w), 1389 (w), 1236 (m), 1214 (w), 1192 (w), 1182 (w), 1156 (w), 1105 (m), 1053 (m), 1045 (m), 1032 (m), 996 (m), 951 (w), 931 (w), 831 (m), 815 (s), 745 (s), 721 (s), 690 (vs), 606 (w), 499 (s), 483 (s) cm^{-1}. ^{1}H NMR (600 MHz, $CDCl_3$): δ = 7.53–7.54 (m, 2 $H_{arom.}$), 7.20–7.22 (m, 3 $H_{arom.}$), 7.16–7.18 (m, 1 $H_{arom.}$), 7.06–7.13 (m, 6 $H_{arom.}$), 6.88 (dd, $J_{H,H}$

= 3.6 Hz, $J_{H,H}$ = 4.8 Hz, 1 $H_{arom.}$), 4.35 (bs, 1 H-Fc), 4.14 (bs, 1 H-Fc), 4.02 (s, 5 H-Fc), 3.96 (bs, 1 H-Fc), 3.87, 3.75 (AB system, $J_{H,H}$ = 9.0 Hz, CH_2), 3.66 (bs, 1H-Fc) ppm. ^{13}C NMR (150 MHz, $CDCl_3$): δ = 147.7, 143.4, 141.3 (3 $C_{arom.}$), 132.2, 130.9, 127.8, 127.2, 126.6, 126.3, 126.1, 125.6, 123.9 (13 $CH_{arom.}$), 92.3, 79.0, 72.7 (C-Fc, 2 C_q), 72.8, 71.9 (2 CH-Fc), 69.5 (5 CH-Fc), 67.6, 67.3 (2 CH-Fc), 30.9 (CH_2) ppm. MS (ESI): *m/z* (%): 524 (35, $[M]^+$), 523 (100, $[M-H]^+$). Anal. calcd. for $C_{29}H_{24}FeS_3$ (524.54): C 66.40, H 4.61, S 18.34; found: C 66.35, H 4.63, S 18.12.

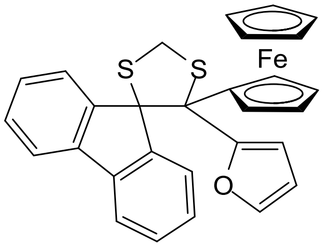

*5-Ferrocenyl-5-(furan-2-yl)spiro[1,3-dithiolane-4,9'-[9*H*]fluorene]* (**5d**): Yield: 167 mg (33%). Yellow crystals; m.p.: ca. 220 °C (decomposition). IR (KBr): ν = 3101 (w), 3047 (w), 2914 (m), 2844 (w), 1955 (w), 1635 (w), 1600 (w), 1575 (w), 1492 (m), 1473 (m), 1445 (s), 1413 (m), 1388 (w), 1367 (w), 1290 (m), 1220 (m), 1185 (m), 1144 (m), 1106 (m), 1052 (m), 1017 (m), 998 (m), 967 (m), 938 (m), 869 (m), 821 (s), 780 (m), 751 (s), 742 (vs), 729 (s), 618 (m), 599 (m), 508 (s) cm^{-1}. ^{1}H NMR (600 MHz, $CDCl_3$): δ = 7.56 (d, $J_{H,H}$ = 7.2 Hz, 2 $H_{arom.}$), 7.41 (d, $J_{H,H}$ = 7.8 Hz, 1 $H_{arom.}$), 7.24–7.29 (m, 2 $H_{arom.}$), 7.11 (t, $J_{H,H}$ = 7.8 Hz, 1 $H_{arom.}$), 7.00–7.02 (m, 2 $H_{arom.}$), 6.64 (d, $J_{H,H}$= 7.8 Hz, 1 $H_{arom.}$), 6.31 (d, $J_{H,H}$ = 3.0 Hz, 1 $H_{arom.}$), 6.13 (dd, $J_{H,H}$ = 1.8 Hz, $J_{H,H}$ = 3.0 Hz, 1 $H_{arom.}$), 4.78 (bs, 1 H-Fc), 4.44, 4.32 (AB system, $J_{H,H}$ = 9.6 Hz CH_2), 4.23 (bs, 1 H-Fc), 3.98 (bs, 1 H-Fc), 3.96 (s, 5 H-Fc), 3.83 (bs, 1 H-Fc) ppm. ^{13}C NMR (150 MHz, $CDCl_3$): δ = 155.1, 146.6, 142.1, 140.7, 139.3 (5 $C_{arom.}$), 140.3, 128.3, 128.0, 127.9, 126.9, 126.1, 125.4, 119.3, 119.1, 109.7, 108.8 (11 $CH_{arom.}$), 92.3, 74.8, 70.3 (C-Fc, 2 C_q), 72.1, 71.2 (2 CH-Fc), 69.0 (5 CH-Fc), 67.8, 67.7 (2 CH-Fc), 32.1 (CH_2) ppm. MS (ESI): *m/z* (%): 507 (33, $[M+H]^+$), 506 (100, $[M]^+$). Anal. calcd. for $C_{29}H_{22}FeOS_2$ (506.46): C 68.77, H 4.38, S 12.66; found: C 68.95, H 4.66, S 12.49.

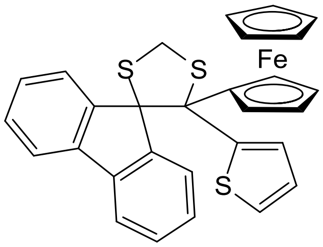

*5-Ferrocenyl-5-(thiophen-2-yl)spiro[1,3-dithiolane-4,9'-[9*H*]fluorene]* (**5e**): Yield: 167 mg (32%). Yellow crystals; m.p.: 87.7–89.5 °C. IR (KBr): ν = 3090 (w), 3058 (w), 2921 (w), 1632 (w), 1474 (w), 1446 (s), 1429 (m), 1390 (w), 1235 (m), 1223 (m), 1108 (m), 1049 (m), 1034 (m), 1001 (m), 852 (w), 837 (w), 816 (m), 755 (vs), 746 (vs), 685 (m), 620 (w), 506 (m), 485 (m) cm^{-1}. ^{1}H NMR (600 MHz, $CDCl_3$): δ = 7.75 (d, $J_{H,H}$ = 7.8 Hz, 1 $H_{arom.}$), 7.63 (d, $J_{H,H}$ = 7.2 Hz, 1 $H_{arom.}$), 7.55 (d, $J_{H,H}$ = 7.8 Hz, 1 $H_{arom.}$), 7.34 (t, $J_{H,H}$ = 7.2 Hz, 1 $H_{arom.}$), 7.19 (t, $J_{H,H}$ = 7.2 Hz, 1 $H_{arom.}$), 7.10 (t, $J_{H,H}$ = 7.2 Hz, 1 $H_{arom.}$), 7.00 (t, $J_{H,H}$ = 7.8 Hz, 1 $H_{arom.}$), 6.85 (d, $J_{H,H}$ = 4.8 Hz, 1 $H_{arom.}$), 6.53 (dd, $J_{H,H}$ = 3.6 Hz, $J_{H,H}$ = 4.8 Hz, 1 $H_{arom.}$), 6.28 (d, $J_{H,H}$ = 7.8 Hz, 1 $H_{arom.}$), 6.18 (d, $J_{H,H}$ = 3Hz, 1 $H_{arom.}$), 4.98 (bs, 1 H-Fc), 4.50, 4.27 (AB system, $J_{H,H}$ = 9.6 Hz, CH_2), 4.46 (bs, 1 H-Fc), 4.09 (bs, 1 H-Fc), 4.07 (s, 5 H-Fc), 3.86 (bs, 1 H-Fc) ppm. ^{13}C NMR (150 MHz, $CDCl_3$): δ = 149.1, 145.0, 141.7, 139.4, 138.3 (5 $C_{arom.}$), 128.8, 128.6, 127.5, 127.3, 126.3, 125.8, 124.9, 124.8, 122.6, 119.5, 119.4 (11 $CH_{arom.}$), 96.2, 75.5, 71.9 (C-Fc, 2 C_q), 73.5, 71.6 (2 CH-Fc), 69.3 (5 CH-Fc), 69.1, 67.4 (2 CH-Fc), 33.0 (CH_2) ppm. MS (ESI): *m/z* (%): 523 (43, $[M+H]^+$), 522 (100, $[M]^{+}$). Anal. calcd. for $C_{29}H_{22}FeS_3$ (522.52): C 66.66, H 4.24, S 18.41; found: C 66.40, H 4.20, S 18.66.

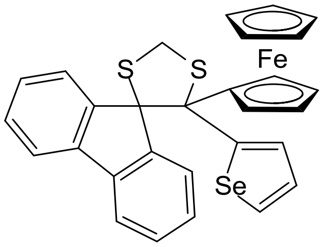

*5-Ferrocenyl-5-(selenophen-2-yl)spiro[1,3-dithiolane-4,9'-[9*H*]fluorene]* (**5f**): Yield: 182 mg (32%). Yellow crystals; m.p.: ca. 192 °C (decomposition). IR (KBr): ν = 3053 (w), 2972 (w), 2920 (w), 2864 (w), 1632 (w), 1601 (w), 1474 (m), 1445 (s), 1234 (m), 1108 (m), 1047 (m), 1034 (m), 1002 (m), 818 (m), 755 (vs), 741 (vs), 674 (m), 511 (m), 500 (m) cm^{-1}. ^{1}H NMR (600 MHz, $CDCl_3$): δ = 7.83 (d, $J_{H,H}$ = 7.8 Hz, 1 $H_{arom.}$),

7.63 (d, $J_{H,H}$ = 7.8 Hz, 1 $H_{arom.}$), 7.53–7.56 (m, 2 $H_{arom.}$), 7.34 (t, $J_{H,H}$ = 7.8 Hz, 1 $H_{arom.}$), 7.19–7.22 (m, 1 $H_{arom.}$), 7.12 (t, $J_{H,H}$ = 7.8 Hz, 1 $H_{arom.}$), 6.99 (t, $J_{H,H}$ = 1.8 Hz, 1 $H_{arom.}$), 6.77 (dd, $J_{H,H}$ = 4.2 Hz, $J_{H,H}$ = 5.4 Hz, 1 $H_{arom.}$), 6.32 (d, $J_{H,H}$ = 3.6 Hz, 1 $H_{arom.}$), 6.28 (d, $J_{H,H}$ = 7.8 Hz, 1 $H_{arom.}$), 4.99 (bs, 1 H-Fc), 4.55, 4.26 (AB system, $J_{H,H}$ = 9.0 Hz, CH_2), 4.48 (bs, 1 H-Fc), 4.09–4.10 (m, 6 H-Fc), 3.92 (bs, 1 H-Fc) ppm. ^{13}C NMR (150 MHz, $CDCl_3$): δ = 149.0, 141.9, 139.3, 138.4 (5 $C_{arom.}$), 128.9, 128.7, 128.6, 128.5, 128.3, 127.5, 127.3, 126.7, 125.3, 119.5, 119.4 (11 $CH_{arom.}$), 95.8, 75.3, 73.7 (C-Fc, 2 C_q), 73.6, 71.7 (2 CH-Fc), 69.4 (5 CH-Fc), 69.2, 67.5 (2 CH-Fc), 33.1 (CH_2) ppm. MS (ESI): *m/z* (%): 571 (32, $[M+2H]^+$), 570 (100, $[M+H]^+$), 568 (53, $[M-H]^+$). Anal. calcd. for $C_{29}H_{22}FeS_2Se$ (569.42): C 61.17, H 3.89, S 11.26; found: C 61.17, H 4.09, S 11.23.

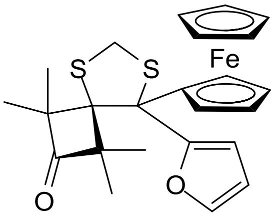

8-Ferrocenyl-8-(furan-2-yl)-1,1,3,3-tetramethyl-5,7-dithiaspiro[3.4]octan-2-one (**5h**): Isolated as the major product. Yield: 289 mg (62%) (crude product ratio 76 : 24). Yellow solid; m.p.: 198.0–199.6 °C. IR (KBr): ν = 3097 (w), 3034 (w), 3008 (m), 2980 (m), 2967 (m), 2924 (m), 2872 (m), 1777 (vs, C=O), 1496 (m), 1456 (s), 1444 (s), 1385 (s), 1378 (s), 1362 (m), 1250 (w), 1212 (s), 1172 (s), 1146 (s), 1107 (s), 1013 (vs), 980 (m), 957 (m), 932 (m), 905 (m), 823 (vs), 791 (s), 778 (s), 731 (vs), 599 (m), 493 (vs) cm^{-1}. ^{1}H NMR (600 MHz, $CDCl_3$): δ = 7.50 (dd, $J_{H,H}$ = 0.6 Hz, $J_{H,H}$ = 1.8 Hz, 1 $H_{arom.}$), 6.80 (dd, $J_{H,H}$ = 3.6 Hz, $J_{H,H}$ = 0.6 Hz, 1 $H_{arom.}$), 6.53 (dd, $J_{H,H}$ = 1.8 Hz, $J_{H,H}$ = 3.6 Hz, 1 $H_{arom.}$), 4.82–4.83 (m, 1 H-Fc), 4.55–4.56 (m, 1 H-Fc), 4.17–4.18 (m, 1 H-Fc), 4.13–4.14 (m, 1 H-Fc), 3.99 (s, 5 H-Fc), 3.71, 3.69 (AB system, $J_{H,H}$ = 8.4 Hz, CH_2), 1.10, 1.26, 1.55, 1.63 (s, 4 CH_3) ppm. ^{13}C NMR (150 MHz, $CDCl_3$): δ = 157.9 (C=O), 140.0, 110.8, 108.4 (3 $CH_{arom.}$), 85.1, 75.1, 69.1, 67.6, 65.8, 64.2 ($C_{arom.}$, C-Fc, 4 C_q), 72.7, 71.7 (2 CH-Fc), 70.0 (5 CH-Fc), 68.1, 65.9 (2 CH-Fc), 27.3 (CH_2), 22.6, 24.0, 24.6, 26.0 (4 CH_3) ppm. MS (ESI): *m/z* (%): 490 (23, $[M+Na+H]^+$), 489 (100, $[M+Na]^+$), 466 (63, $[M]^+$). Anal. calcd. for $C_{24}H_{26}FeO_2S_2$ (466.44): C 61.80, H 5.62, S 13.75; found: C 61.85, H 5.85, S 13.70.

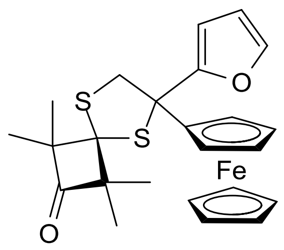

6-Ferrocenyl-6-(furan-2-yl)-1,1,3,3-tetramethyl-5,8-dithiaspiro[3.4]octan-2-one (**6h**) (from the spectra of a mixture of **6h** with the major product **5h**): ^{1}H NMR (600 MHz, $CDCl_3$): δ = 7.48 (bs, 1 $H_{arom.}$), 6.56 (bs, 1 $H_{arom.}$), 6.42 (bs, 1 $H_{arom.}$), 4.39 (bs, 1 H-Fc), 4.19 (bs, 1 H-Fc), 4.18 (s, 6 H-Fc), 4.02 (bs, 1 H-Fc), 3.73, 3.47 (AB system, $J_{H,H}$ = 12.0 Hz, CH_2), 1.42, 1.41, 1.28, 1.16 (s, 4 CH_3) ppm. ^{13}C NMR (150 MHz, $CDCl_3$): δ = 156.4 (C=O), 141.3, 108.5, 101.2 (3$CH_{arom.}$), 90.6, 75.3, 66.6, 66.4, 66.3 ($C_{arom.}$, C-Fc, 4 C_q), 69.2, 68.0, 67.9, 67.4, 67.3 (9 CH-Fc), 49.3 (CH_2), 24.3, 22.9, 22.2, 21.0 (4 CH_3) ppm.

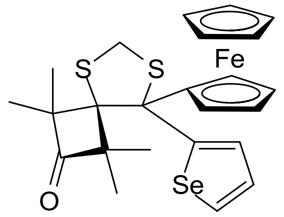

8-Ferrocenyl-1,1,3,3-tetramethyl-8-(selenophen-2-yl)-5,7-dithiaspiro[3.4]octan-2-one (**5j**): Isolated as the major product. Yield: 280 mg (53%) (crude product ratio 78 : 22). Orange crystals; m.p.: ca. 178 °C (decomposition). IR (KBr): ν = 3133 (w), 3114 (w), 3094 (w), 3042 (w), 3022 (m), 2970 (m), 2931 (m), 2867 (m), 1774 (vs, C=O), 1469 (m), 1455 (m), 1385 (m), 1363 (m), 1229 (s), 1166 (m), 1106 (m), 1066 (m), 1022 (m), 1003 (m), 901 (m), 823 (s), 778 (m), 757 (m), 681 (vs), 490 (s) cm^{-1}. ^{1}H NMR (600 MHz, $CDCl_3$): δ = 7.98 (d, $J_{H,H}$ = 5.4 Hz, 1 $H_{arom.}$), 7.36–7.39 (m, 2 $H_{arom.}$), 4.89 (bs, 1 H-Fc), 4.49 (bs, 1 H-Fc), 4.26 (bs, 1 H-Fc), 4.17 (bs, 1 H-Fc), 3.98 (s, 5 H-Fc), 3.83, 3.79 (AB system, $J_{H,H}$ = 8.4 Hz, CH_2), 1.70, 1.63 (s, 2 CH_3), 1.18, 1.17 (bs, 2 CH_3) ppm. ^{13}C NMR (150 MHz, $CDCl_3$): δ = 162.8 (C=O) 129.8, 129.5, 123.8 (3 $CH_{arom.}$), 84.3, 76.4, 72.5, 71.3, 65.8, 62.8 ($C_{arom.}$, C-Fc, 4 C_q), 72.8, 72.2 (2 CH-Fc), 70.3 (5 CH-Fc), 68.7, 66.4 (2 CH-Fc), 28.9 (CH_2), 26.2, 25.0, 23.8, 23.4 (4 CH_3) ppm. MS (ESI): *m/z* (%): 531 (100, $[M+2H]^+$), 529 (65, $[M]^+$). Anal. calcd. for $C_{24}H_{26}FeOS_2Se$ (529.40): C 54.45, H 4.95, S 12.11; found: C 54.52, H 5.14, S 12.01.

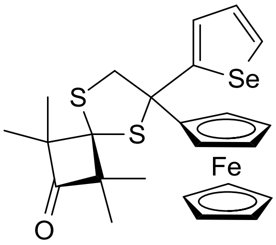

6-Ferrocenyl-1,1,3,3-tetramethyl-6-(selenophen-2-yl)-5,8-dithiaspiro[3.4]octan-2-one (**6j**) (from the spectra of a mixture of **6j** with the major product **5j**): ^{1}H NMR (600 MHz, CDCl$_3$): δ = 7.93 (dd, $J_{H,H}$ = 1.2 Hz, $J_{H,H}$ = 5.4 Hz, 1 H$_{arom.}$), 7.28–7.29 (m, 1 H$_{arom.}$), 7.25 (dd, $J_{H,H}$ = 1.2 Hz, $J_{H,H}$ = 4.2 Hz, 1 H$_{arom.}$), 4.35–4.36 (m, 1 H-Fc), 4.23–4.24 (m, 1 H-Fc), 4.22 (s, 5 H-Fc), 4.15–4.16 (m, 1 H-Fc), 4.09–4.10 (m, 1 H-Fc), 3.66, 3.54 (AB system, $J_{H,H}$ = 12.0 Hz, CH$_2$), 1.49, 1.46, 1.44, 1.16 (s, 4 CH$_3$) ppm. ^{13}C NMR (150 MHz, CDCl$_3$): δ = 159.2 (C=O), 130.2, 129.3, 126.7 (3 CH$_{arom.}$), 92.9, 75.4, 71.0, 67.4, 66.2 (C$_{arom.}$, C-Fc, 4 C$_q$), 69.4, 68.5, 68.2, 68.0, 67.4 (9 CH-Fc), 53.9 (CH$_2$), 25.3, 24.6, 22.6, 22.0 (4 CH$_3$) ppm.

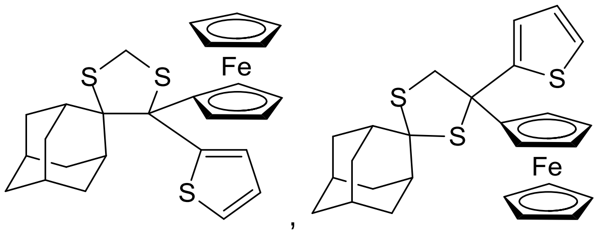

5-Ferrocenyl-5-(thiophen-2-yl)spiro[1,3-dithiolane-4,2'-tricyclo[3.3.1.1^{3,7}]-decane] (**5k**);*4-Ferrocenyl-4-(thiophen-2-yl)-spiro[1,3-dithiolane-2,2'-tricyclo[3.3.1.1^{3,7}]-decane]* (**6k**): Isolated as a mixture of regioisomers. Yield: 280 mg (57%) (crude product ratio 75 : 25). Yellow solid. IR (KBr): ν = 3088 (w), 2901 (vs), 2847 (s), 1635 (w), 1451 (m), 1423 (m), 1391 (m), 1347 (m), 1252 (w), 1230 (m), 1211 (m), 1100 (m), 1027 (m), 1002 (m), 960 (m), 929 (m), 821 (m), 812 (m), 755 (m), 710 (s), 482 (s) cm^{-1}. ^{1}H NMR (600 MHz, CDCl$_3$): δ = 7.27 (d, $J_{H,H}$ = 5.2 Hz, 1 H$_{arom.}$), 7.21 (d, $J_{H,H}$ = 5.1 Hz, 1 H$_{arom.}$), 7.09–7.12 (m, 2 H$_{arom.}$), 7.03–7.04 (m, 1 H$_{arom.}$), 6.98 (t, $J_{H,H}$ = 4.0 Hz, 1 H$_{arom.}$), 4.93 (bs, 1 H-Fc), 4.27–4.28 (m, 1 H-Fc), 4.18–4.20 (m, 6 H-Fc), 4.16–4.17 (m, 1 H-Fc), 4.08 (bs, 1 H-Fc), 3.98 (s, 5 H-Fc), 3.93–3.94 (m, 1 H-Fc), 3.87, 3.63 (AB system, $J_{H,H}$ = 8.4 Hz, CH$_2$ (major)), 3.82, 3.60 (AB system, $J_{H,H}$ = 12.0 Hz, CH$_2$ (minor)), 2.93 (d, $J_{H,H}$ = 11.9 Hz, 1 H$_{alif.}$), 1.22–2.53 (m, 27 H$_{alif.}$) ppm. ^{13}C NMR (150 MHz, CDCl$_3$): δ = 152.0, 151.9 (2 C$_{arom.}$), 126.6, 126.4, 125.3, 124.3, 123.9, 123.4 (6 CH$_{arom.}$), 93.8, 88.2 (2 C-Fc), 77.8, 75.0, 73.8, 68.0 (4 C$_q$), 72.4, 71.4, 69.9,

69.4, 69.0, 68.1, 67.9, 67.8, 67.6, 67.3 (18 CH-Fc), 51.9, 40.4, 39.1, 37.7, 37.6, 37.2, 35.9, 35.8, 33.6, 33.2, 25.7 (12 CH_2), 42.8, 41.8, 34.9, 34.3, 27.2, 27.1, 26.4, 26.3 (10 $CH_{alif.}$) ppm. MS (ESI): *m/z* (%): 493 (28, $[M+H]^+$), 492 (100, $[M]^+$). Anal. calcd. for $C_{26}H_{28}FeS_3$ (492.54): C 63.40, H 5.73, S 19.53, found; C 63.47, H 5.92, S 19.38.

2. X-ray crystallography

Crystal data for **5b**: $C_{29}H_{24}FeS_3$, M_r = 524.51, orange, prism, 0.16 × 0.17 × 0.26 mm, T = 160(1) K, orthorhombic, $Pna2_1$, Z = 4, a = 18.26095(19), b = 12.56563(12), c = 9.99949(9) Å, V = 2294.49(4) Å^3, D_x = 1.518 g cm^{-3}, μ(Mo $K\alpha$) = 0.947 mm^{-1}, ω scans, $2\theta_{(max)}$ = 60.9°, transmission factors (min; max) = 0.982; 1.000, 28620 reflections measured, 6342 symmetry independent reflections, 6057 reflections with $I > 2\sigma(I)$, 6342 reflections used in refinement, 299 parameters refined, 1 restraint, $R(F)$ [$I > 2\sigma(I)$ reflections] = 0.0253, $wR(F^2)$ (all data) = 0.0622, $w = [\sigma^2(F_o^2) + (0.0317P)^2 + 0.5668P]^{-1}$ where $P = (F_o^2 + 2F_c^2)/3$, goodness of fit = 1.061, final Δ_{max}/σ = 0.001, $\Delta\rho$(max; min) = 0.41; –0.32 e Å^{-3}. Crystals from hexane/CH_2Cl_2.

Crystal data for **5e**: $C_{29}H_{22}FeS_3$, M_r = 522.49, red, prism, 0.18 × 0.23 × 0.26 mm, T = 160(1) K, monoclinic, $P2_1/c$, Z = 4, a = 9.81475(12), b = 9.36819(14), c = 24.5497(3) Å, β = 93.2593(12)°, V = 2253.61(5) Å^3, D_x = 1.540 g cm^{-3}, μ(Mo $K\alpha$) = 0.964 mm^{-1}, ω scans, $2\theta_{(max)}$ = 61.0°, transmission factors (min; max) = 0.900; 1.000, 28552 reflections measured, 6235 symmetry independent reflections, 5440 reflections with $I > 2\sigma(I)$, 6235 reflections used in refinement, 317 parameters refined, 28 restraints, $R(F)$ [$I > 2\sigma(I)$ reflections] = 0.0313, $wR(F^2)$ (all data) = 0.0797, $w = [\sigma^2(F_o^2) + (0.0351P)^2 + 1.5455P]^{-1}$ where $P = (F_o^2 + 2F_c^2)/3$, goodness of fit = 1.033, final Δ_{max}/σ = 0.002, $\Delta\rho$(max; min) = 0.57; –0.46 e Å^{-3}. Crystals from hexane/CH_2Cl_2.

Crystal data for **5f**: $C_{29}H_{22}FeS_2Se$, M_r = 569.39, orange, plate, 0.06 × 0.15 × 0.24 mm, T = 160(1) K, monoclinic, $P2_1/c$, Z = 4, a = 9.76095(19), b = 9.44484(14), c = 24.6230(4) Å, β = 93.0235(17)°, V = 2266.85(7) Å^3, D_x = 1.668 g cm^{-3}, μ(Mo $K\alpha$) = 2.472 mm^{-1}, ω scans, $2\theta_{(max)}$ = 60.8°, transmission factors (min; max) = 0.777; 1.000, 28927 reflections measured, 6316 symmetry independent reflections, 4870 reflections with $I > 2\sigma(I)$, 6316 reflections used in refinement, 299 parameters refined, $R(F)$ [$I > 2\sigma(I)$ reflections] = 0.0397, $wR(F^2)$ (all data) = 0.0917, $w = [\sigma^2(F_o^2)$ +

$(0.0368P)^2 + 1.8728P]^{-1}$ where $P = (F_o^2 + 2F_c^2)/3$, goodness of fit = 1.038, final Δ_{max}/σ = 0.002, $\Delta\rho$(max; min) = 0.76; –1.00 e Å^{-3}. Crystals from hexane/CH_2Cl_2.

Crystal data for **5g**: $C_{31}H_{24}FeS_2$, M_r = 516.47, red, plate, 0.04 × 0.18 × 0.20 mm, T = 160(1) K, monoclinic, $P2_1/c$, Z = 4, a = 9.77370(19), b = 9.43473(19), c = 25.0337(5) Å, β = 93.6894(17)°, V = 2303.63(8) Å^3, D_x = 1.489 g cm^{-3}, μ(Mo $K\alpha$) = 0.855 mm^{-1}, ω scans, $2\theta_{(max)}$ = 58.6°, transmission factors (min; max) = 0.878; 1.000, 25403 reflections measured, 5604 symmetry independent reflections, 4437 reflections with $I > 2\sigma(I)$, 5604 reflections used in refinement, 307 parameters refined, $R(F)$ [$I > 2\sigma(I)$ reflections] = 0.0391, $wR(F^2)$ (all data) = 0.0965, $w = [\sigma^2(F_o^2) + (0.0431P)^2 + 1.2909P]^{-1}$ where $P = (F_o^2 + 2F_c^2)/3$, goodness of fit = 1.030, final Δ_{max}/σ = 0.001, $\Delta\rho$(max; min) = 0.55; –0.38 e Å^{-3}. Crystals from hexane/CH_2Cl_2

All measurements were made on a Rigaku Oxford Diffraction (formerly Agilent Technologies) SuperNova area-detector diffractometer [S1, S2], using MoKα radiation (λ = 0.71073 Å) from a micro-focus X-ray source and an Oxford Instruments Cryojet XL cooler. Data reduction was performed with CrysAlisPro [S1, S2]. The intensities were corrected for Lorentz and polarization effects, and empirical absorption corrections using spherical harmonics were applied [S1, S2]. Equivalent reflections, other than Friedel pairs in the case of **5b**, were merged. The data collection and refinement parameters are given above, and views of the molecules **5b** and **5f** are shown in **Figure 1** and those of **5e** and **5g** in **Figures S33** and **S34**. The structures were solved by direct methods using SHELXS-2013 [S3], which revealed the positions of all non-H-atoms. In the case of **5e**, the thiophene ring is disordered over two orientations related by a 180° flip of the ring. Two sets of overlapping positions were defined for the S-atom and the corresponding –CH– group on the other side of the ring, and the site occupation factor of the major conformation of the ring refined to 0.875(2). Similarity restaints were applied to the chemically equivalent bond lengths involving all disordered atoms, while neighboring disordered atoms were restrained to have similar and pseudo-isotropic atomic displacement parameters. Similarly, in the case of **5f**, the selenophene ring is disordered by a 180° rotation about its bonding axis. The 2- and 5-positions of the ring were defined as a combination of contributions from a C- and an Se-atom at the same site. The site occupation factor of the major conformation refined to 0.9119(12).

Although the C–C and C–Se bond lengths to these sites are quite distinct, attempts to refine the disordered sites with split positions for the atoms were unsuccessful. Therefore, in the final refinements the sites were treated as mixed site with the C- and Se-atom coordinates and atomic displacement parameters constrained to be identical. The final bond lengths involving disordered atoms correspond closely with those expected for the major occupant of each site. The non-H-atoms of all compounds were refined anisotropically. All of the H-atoms were placed in geometrically calculated positions and refined by using a riding model where each H-atom was assigned a fixed isotropic displacement parameter with a value equal to 1.2Ueq of its parent C-atom). The refinement of each structure was carried out on F^2 by using full-matrix least-squares procedures, which minimized the function $\Sigma w(F_o^2 - F_c^2)^2$. Corrections for secondary extinction were not applied. Refinement of the absolute structure parameter [S4] of **5b** yielded a value of 0.13(1), which indicates that the crystal is a partial inversion twin with a major twin fraction of 0.87(1). Neutral atom scattering factors for non-H-atoms were taken from ref. [S5], and the scattering factors for H-atoms were taken from ref. [S6]. Anomalous dispersion effects were included in F_c; [S7] the values for f' and f'' were those of ref. [S8]. The values of the mass attenuation coefficients are those of ref. [S9]. The *SHELXL-2014* program [S10] was used for all calculations. CCDC-1469435 – 1469348 contain the supplementary crystallographic data for this paper. These data can be obtained free of charge from The *Cambridge Crystallographic Data Centre*, *via* www.ccdc.cam.ac.uk/getstructures.

References

[S1] *CrysAlisPro*, Version 1.171.37.35g (**5b**, **5f**), Agilent Technologies, Yarnton, Oxfordshire, England, 2014,

[S2] *CrysAlisPro*, Version 1.171.38.41 (**5e**, **5g**), Rigaku Oxford Diffraction, Abingdon, Oxfordshire, England, 2015.

[S3] Sheldrick, G. M. *Acta Crystallogr. Sect. A*, **2008**, *64*, 112–122.

[S4] Flack, H. D.; Bernardinelli, G. *Acta Crystallogr., Sect. A,* **1999**, *55*, 908–915; Flack, H. D.; Bernardinelli, G. *J. appl. Crystallogr.* **2000**, *33*, 1143–1148.

[S5] Maslen, E. N.; Fox, A. G.; O'Keefe, M. A. in 'International Tables for Crystallography', Ed. Wilson, A. J. C. Kluwer Academic Publishers, Dordrecht, **1992**, Vol. C, Table 6.1.1.1, pp. 477–486.

[S6] Stewart, R. F.; Davidson, E. R.; Simpson, W. T. *J. Chem. Phys.* **1965**, *42*, 3175–3187. doi: 10.1063/1.1696397.

[S7] Ibers, J. A.; Hamilton, W. C. *Acta Crystallogr.* **1964**, *17*, 781–782. doi: 10.1107/S0365110X64002067.

[S8] Creagh, D. C.; McAuley, W. J. in 'International Tables for Crystallography', Ed. Wilson, A. J. C. Kluwer Academic Publishers, Dordrecht, **1992**, Vol. C, Table 4.2.6.8, pp. 219–222.

[S9] Creagh, D. C.; Hubbell, J. H. in 'International Tables for Crystallography', Ed. Wilson, A. J. C. Kluwer Academic Publishers, Dordrecht, **1992**, Vol. C, Table 4.2.4.3, pp. 200–206.

[S10] Sheldrick, G. M. *Acta Crystallogr. Sect. C*, **2015**, *71*, 3–8. doi: 10.1107/S2053229614024218.

[S11] Johnson, C. K. ORTEP II, Report ORNL-5138, Oak Ridge National Laboratory, Oak Ridge, Tennessee, **1976**.

3. Collection of the ^{1}H and ^{13}C NMR for the described compounds 5, 6

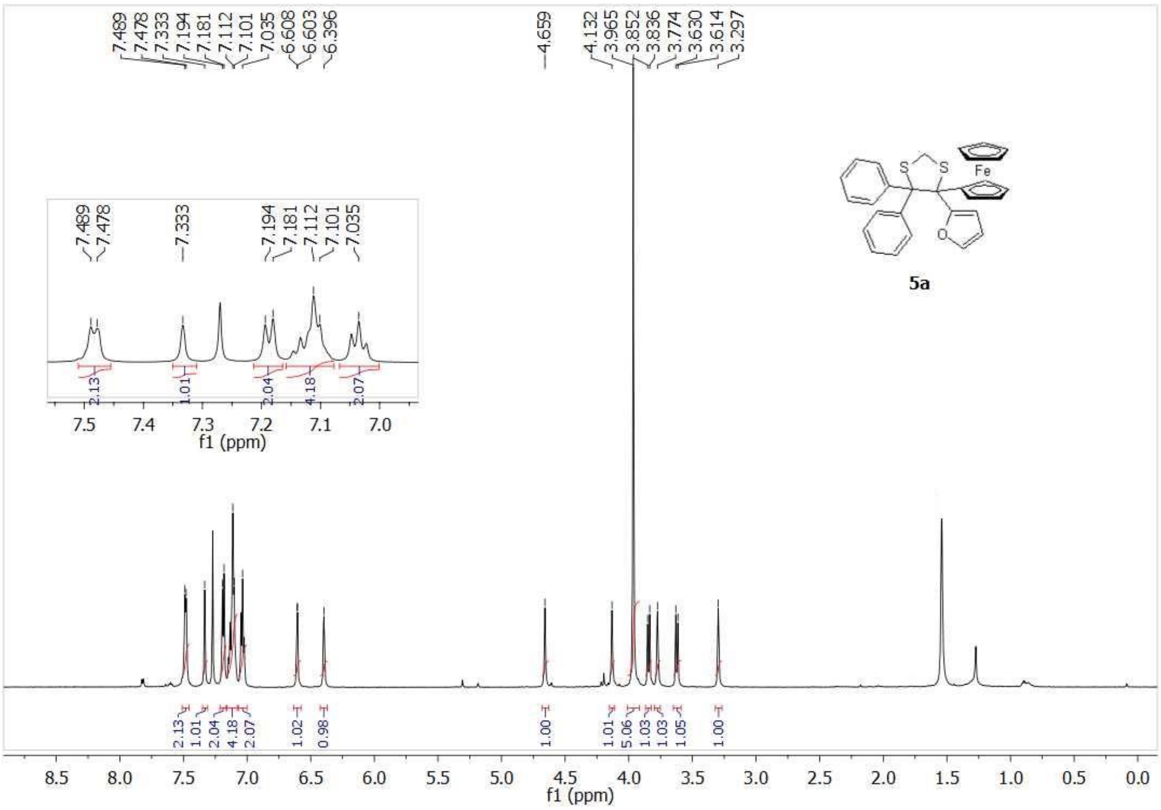

Figure S1. The ^{1}H NMR spectrum of compound **5a**.

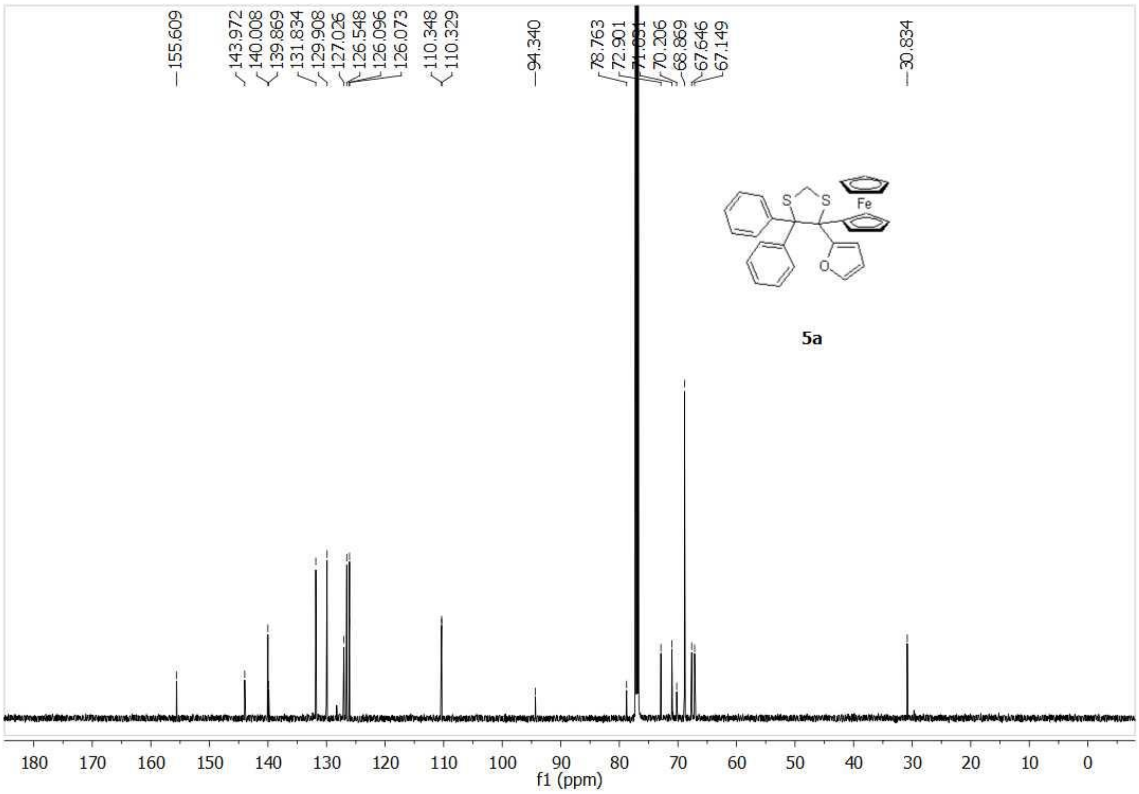

Figure S2. The ^{13}C NMR spectrum of compound **5a**.

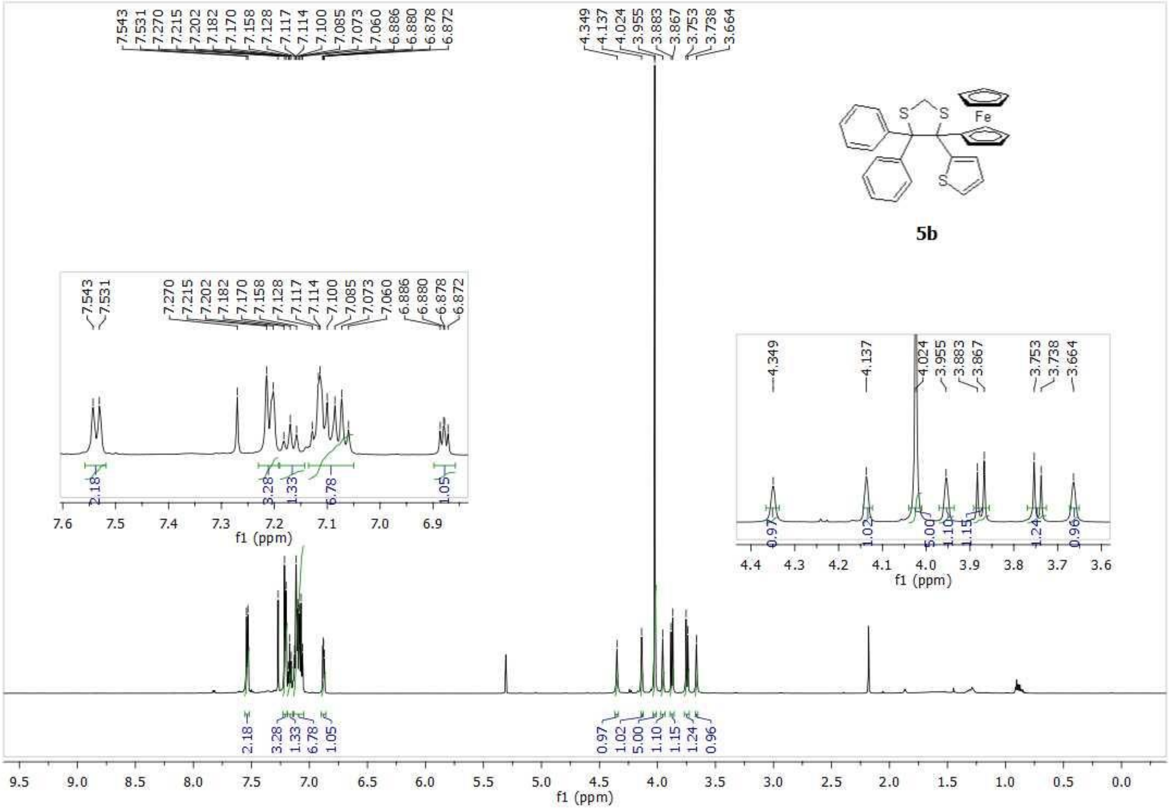

Figure S3. The ^{1}H NMR spectrum of compound **5b**.

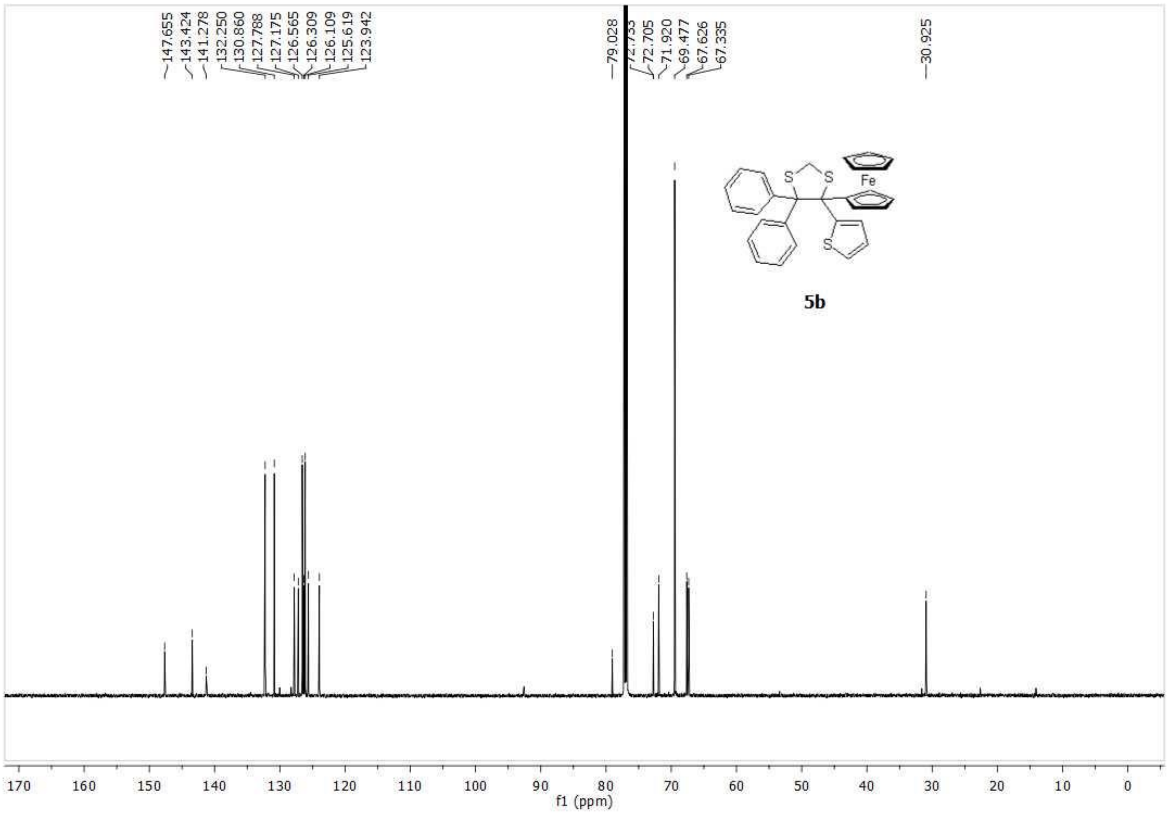

Figure S4. The ^{13}C NMR spectrum of compound **5b**.

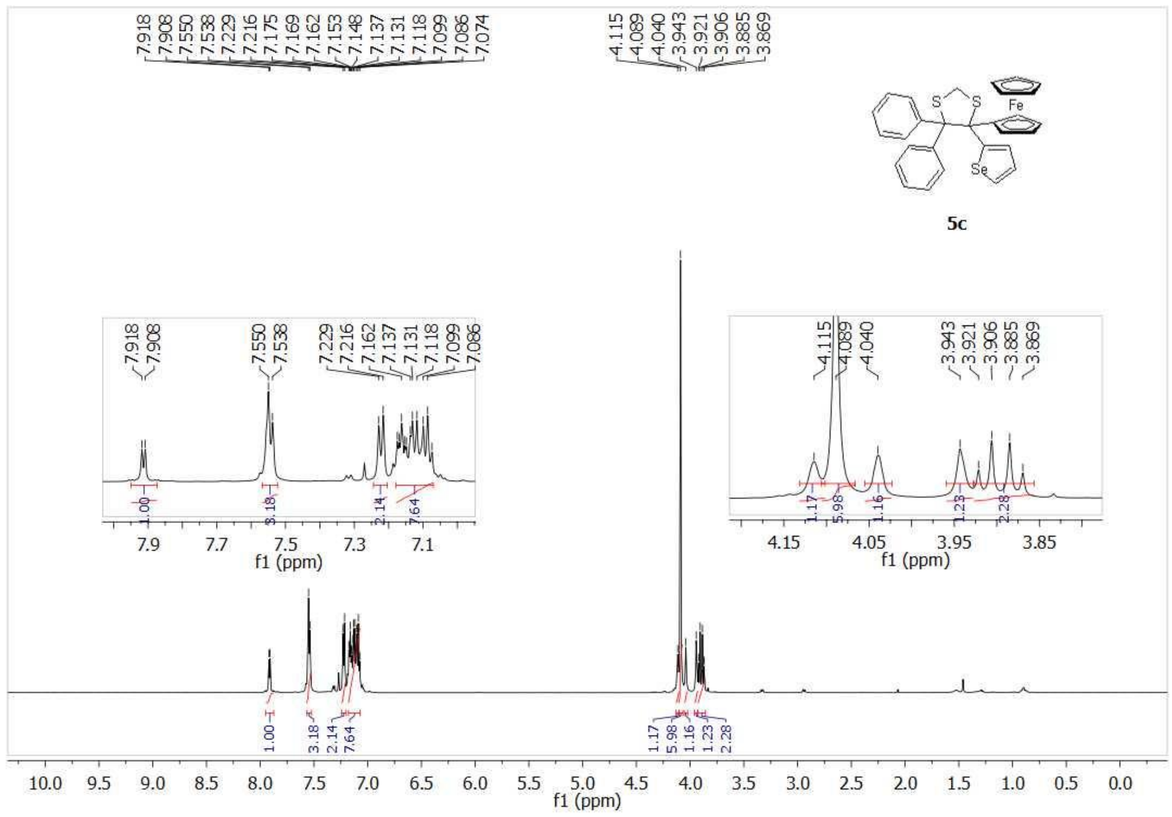

Figure S5. The ^{1}H NMR spectrum of compound **5c**.

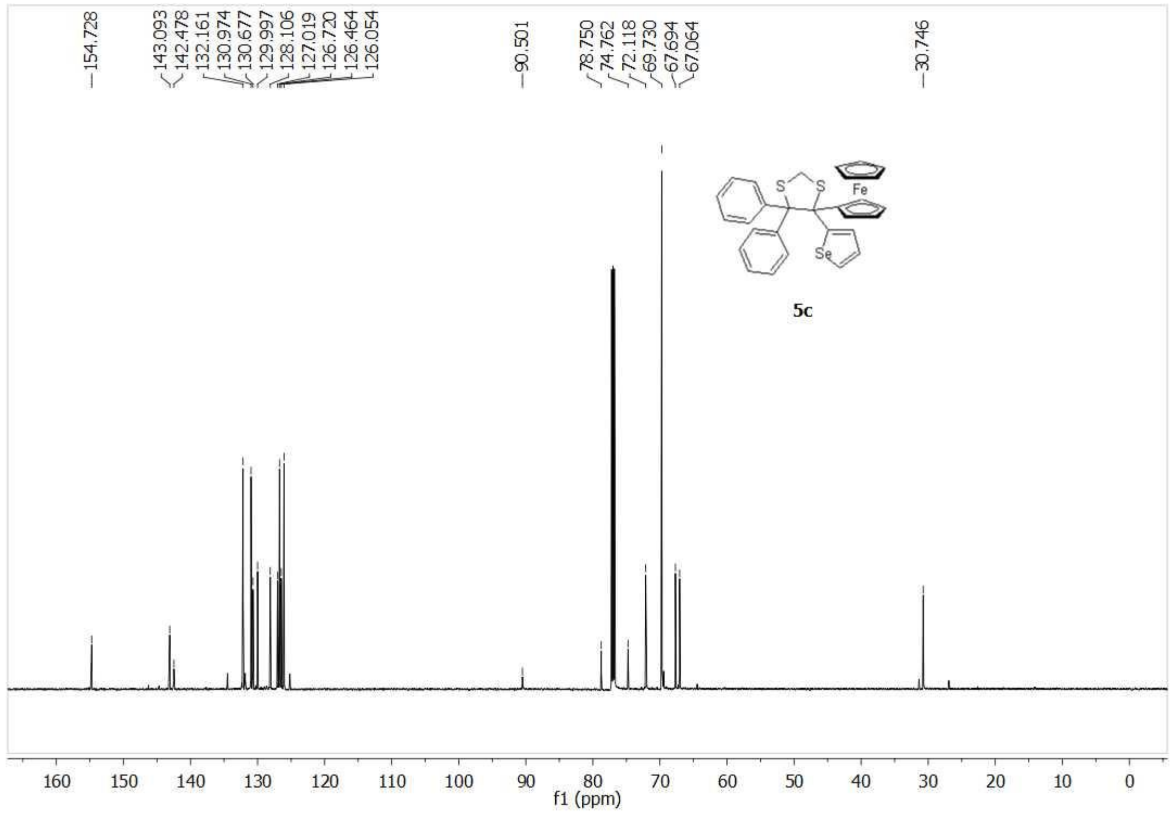

Figure S6. The ^{13}C NMR spectrum of compound **5c**.

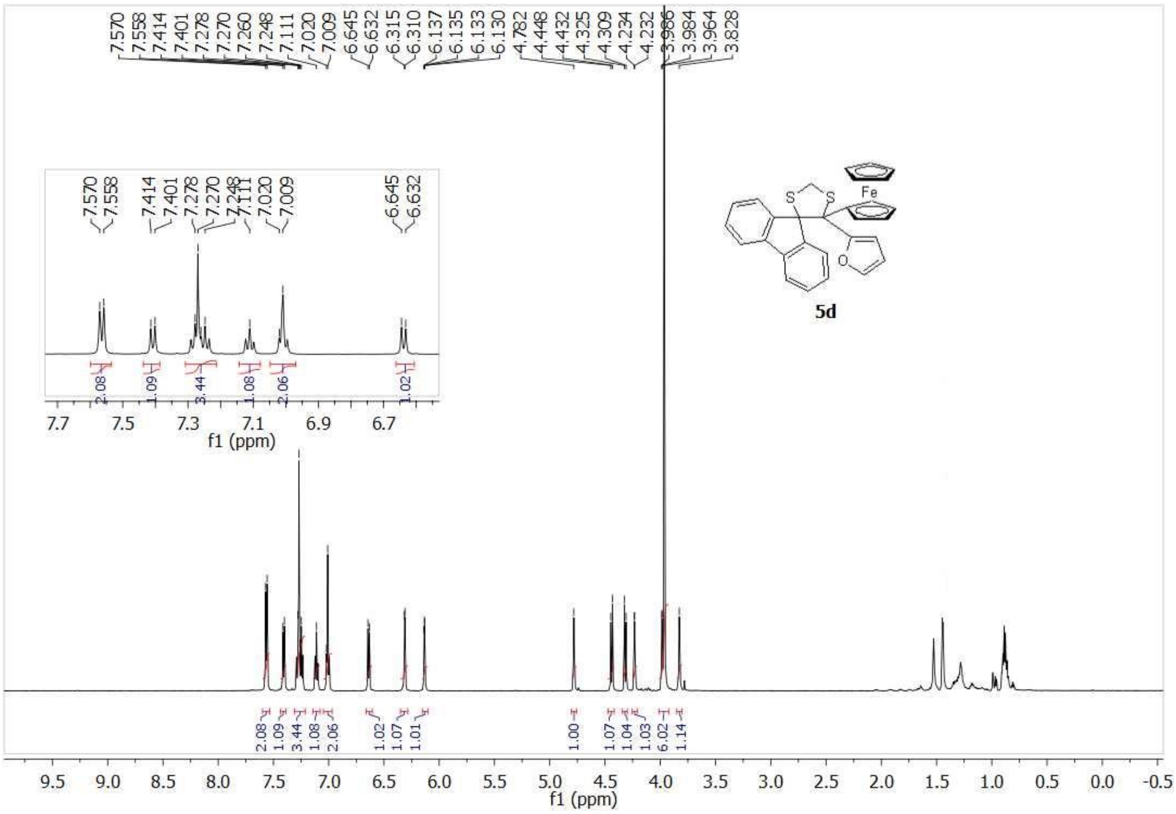

Figure S7. The ^{1}H NMR spectrum of compound **5d**.

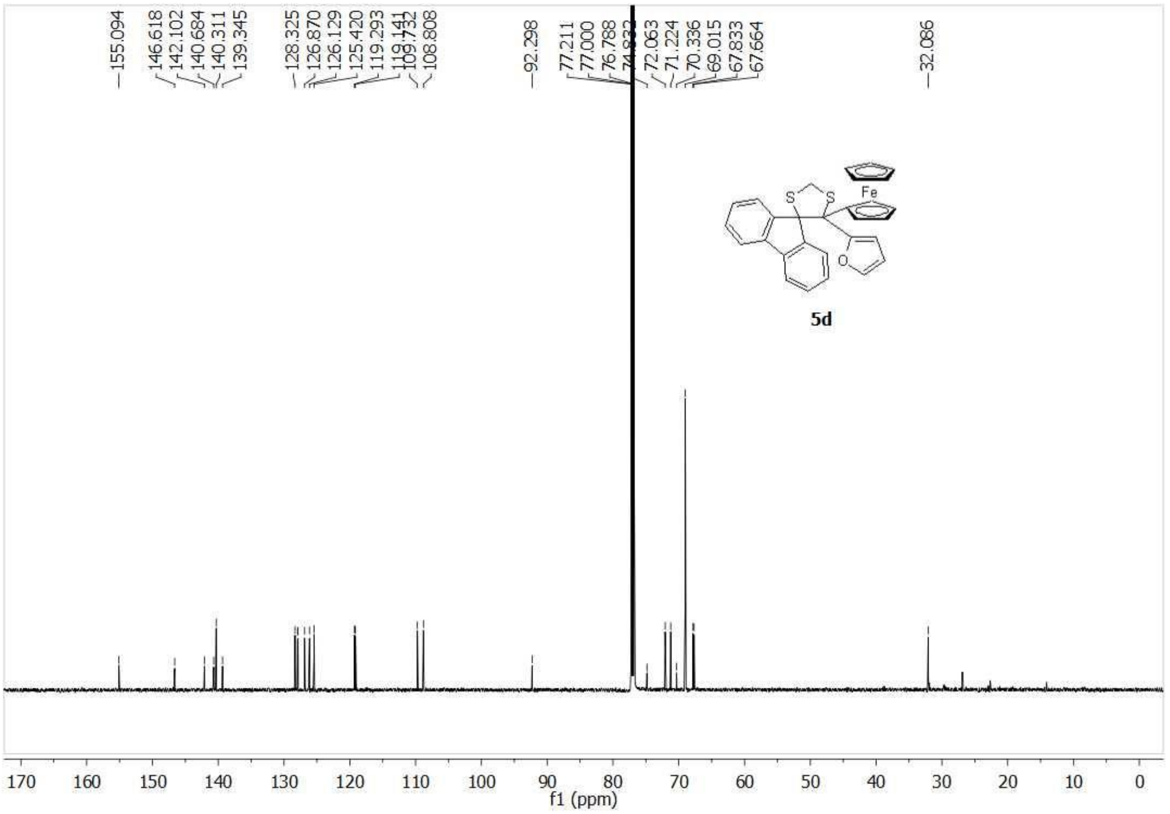

Figure S8. The ^{13}C NMR spectrum of compound **5d**.

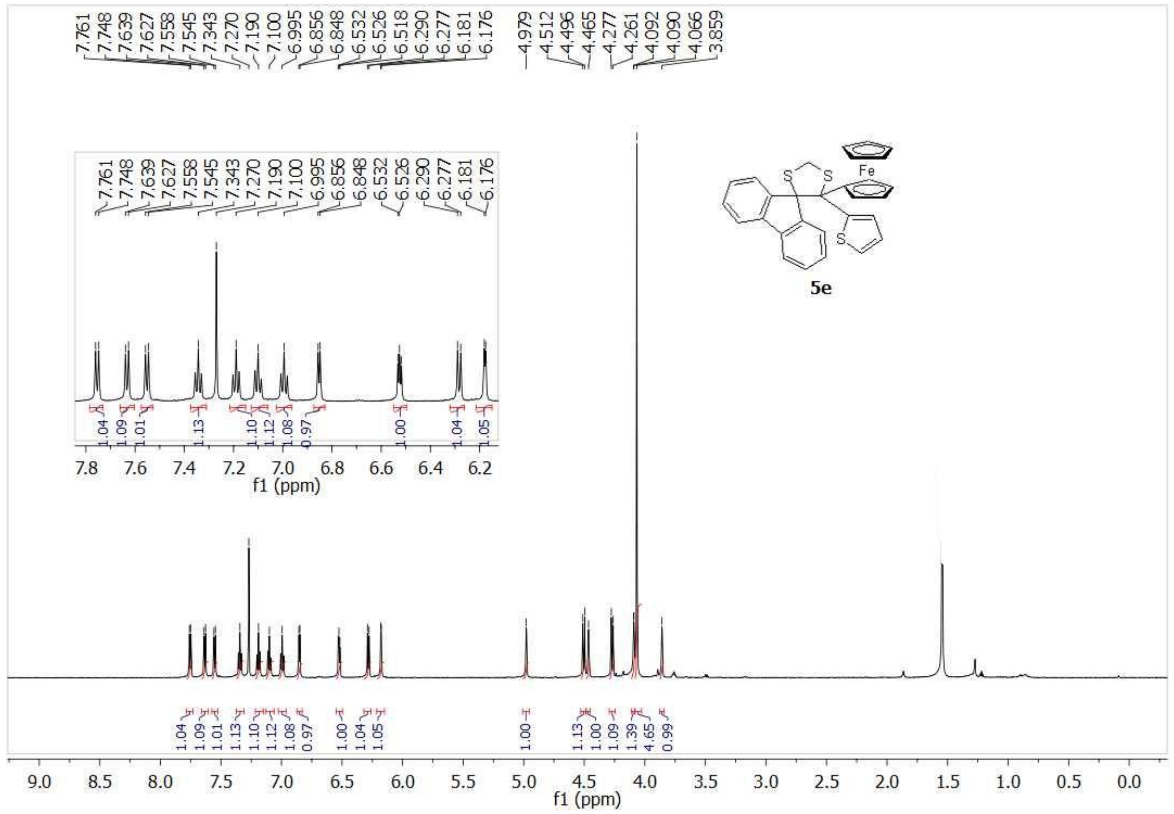

Figure S9. The ^{1}H NMR spectrum of compound **5e**.

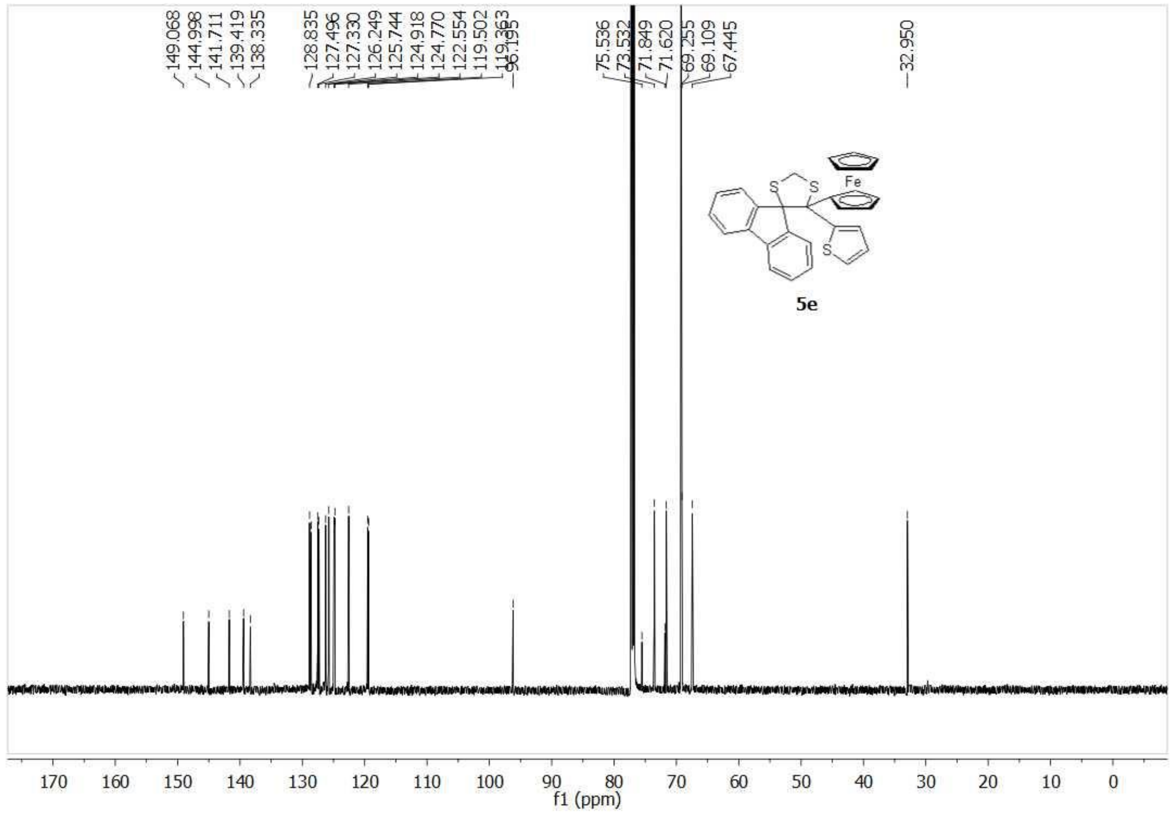

Figure S10. The ^{13}C NMR spectrum of compound **5e**.

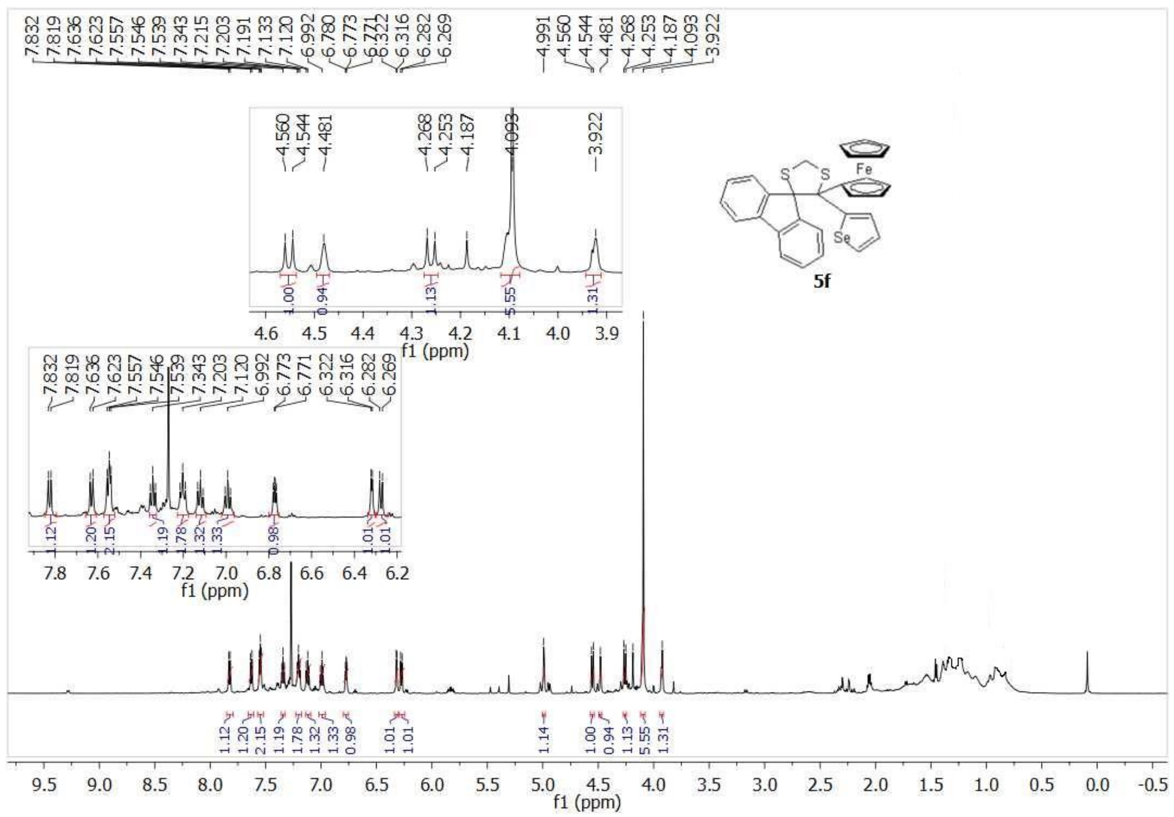

Figure S11. The ^{1}H NMR spectrum of compound **5f.**

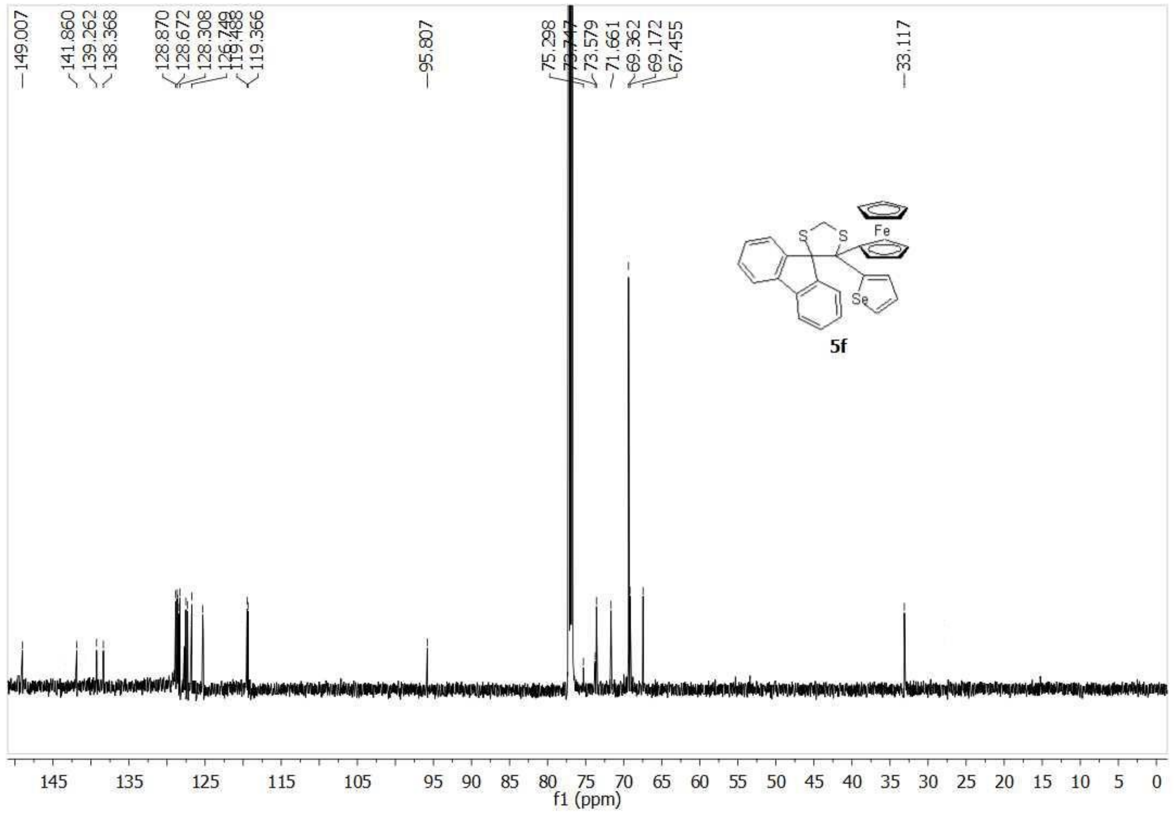

Figure S12. The ^{13}C NMR spectrum of compound **5f**.

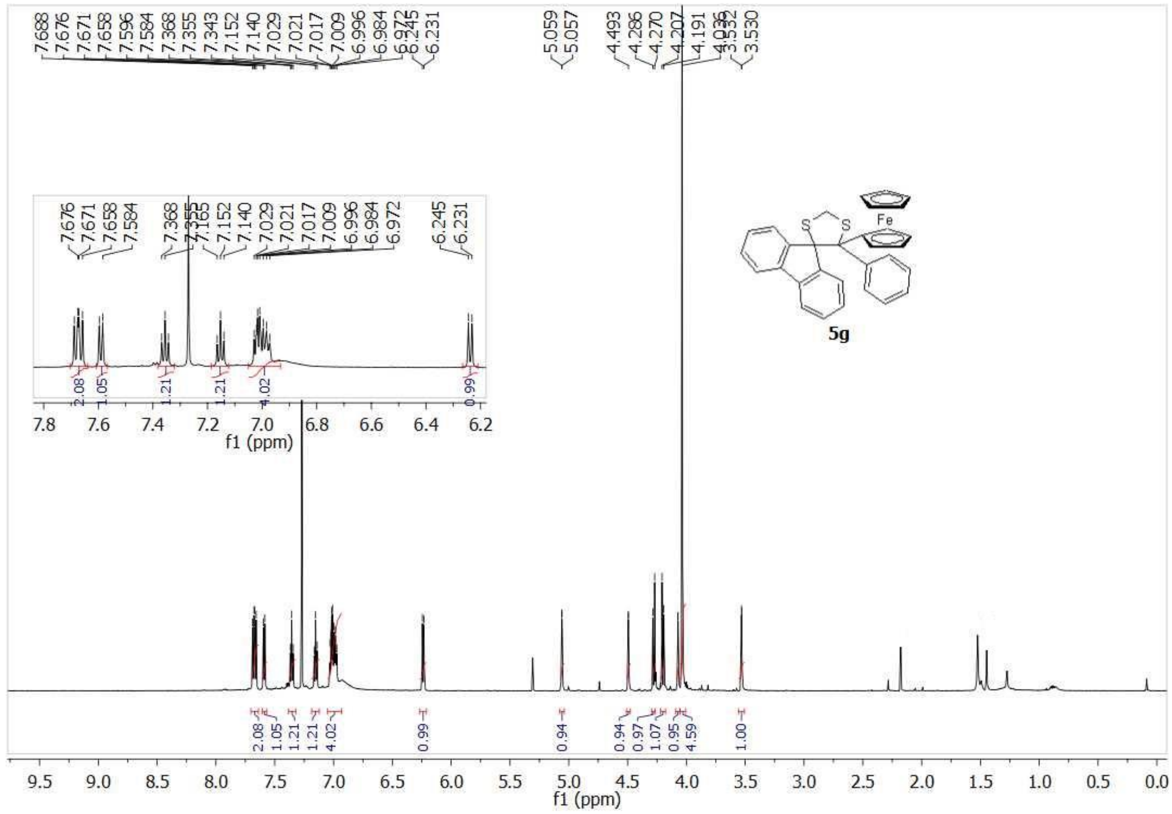

Figure S13. The ^{1}H NMR spectrum of compound **5g**.

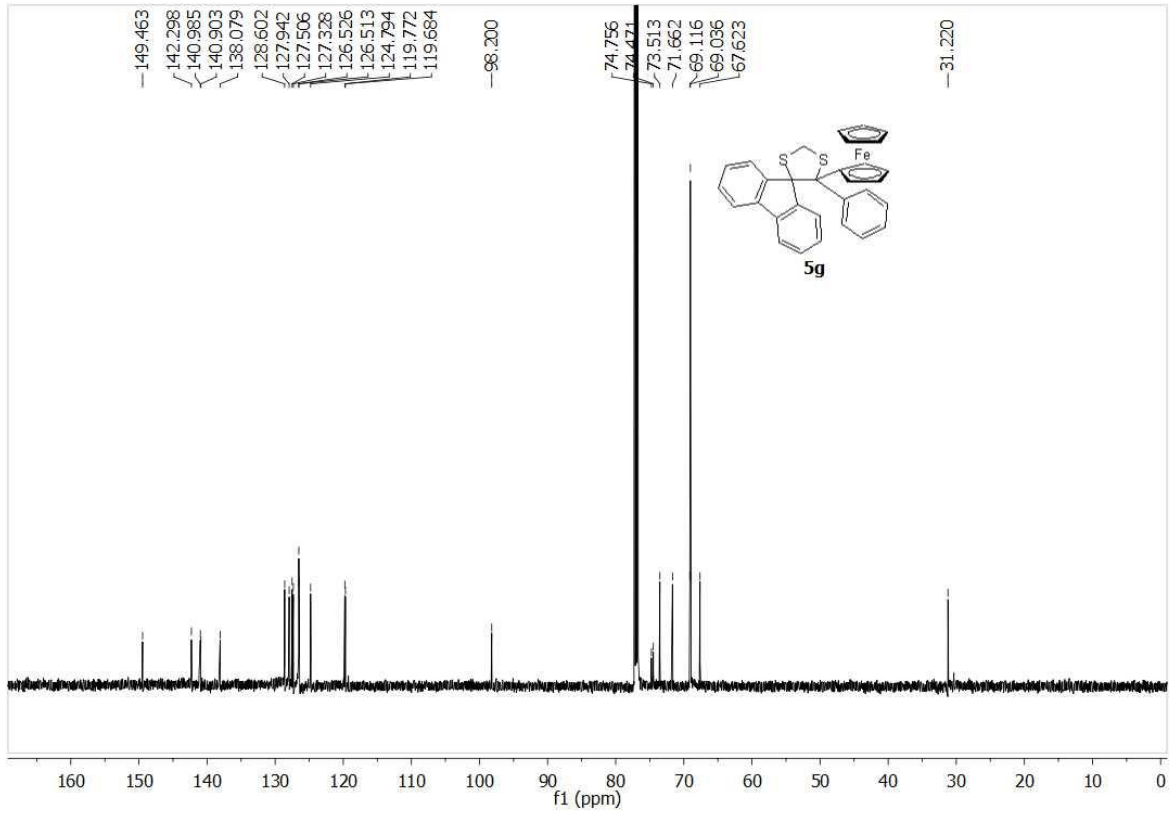

Figure S14. The ^{13}C NMR spectrum of compound **5g**.

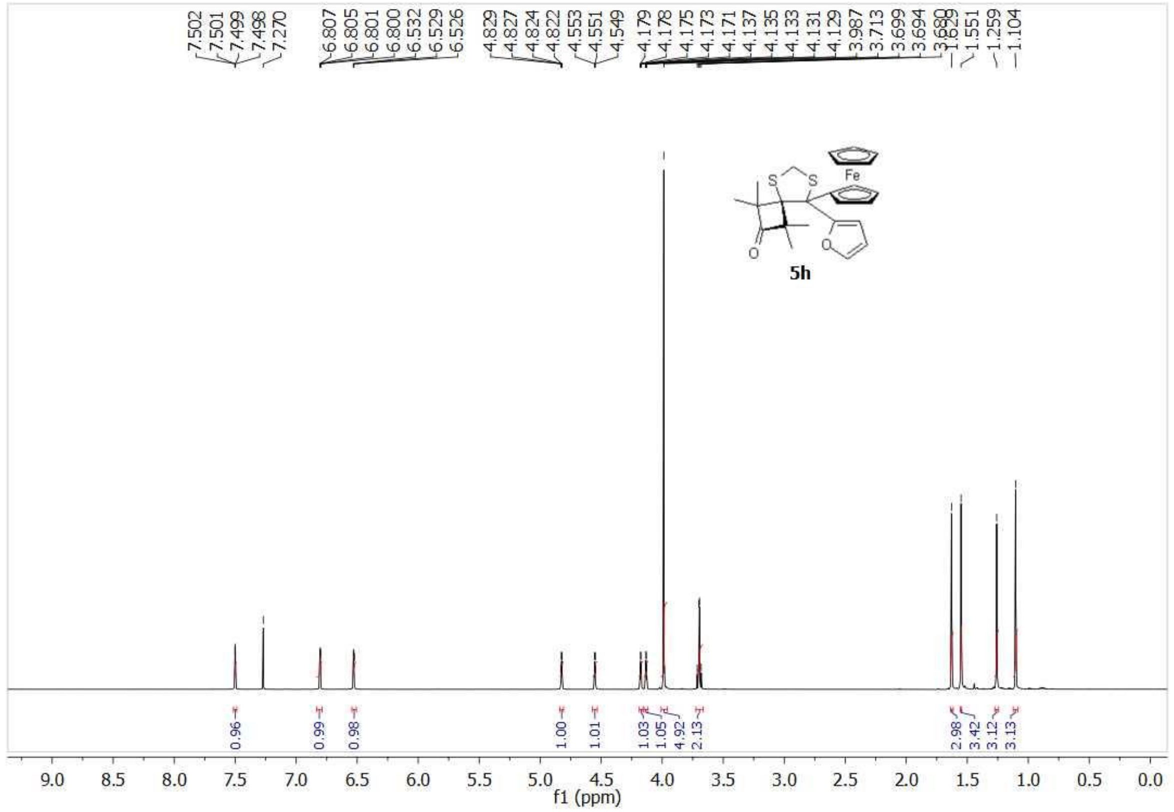

Figure S15. The ^{1}H NMR spectrum of compound **5h**.

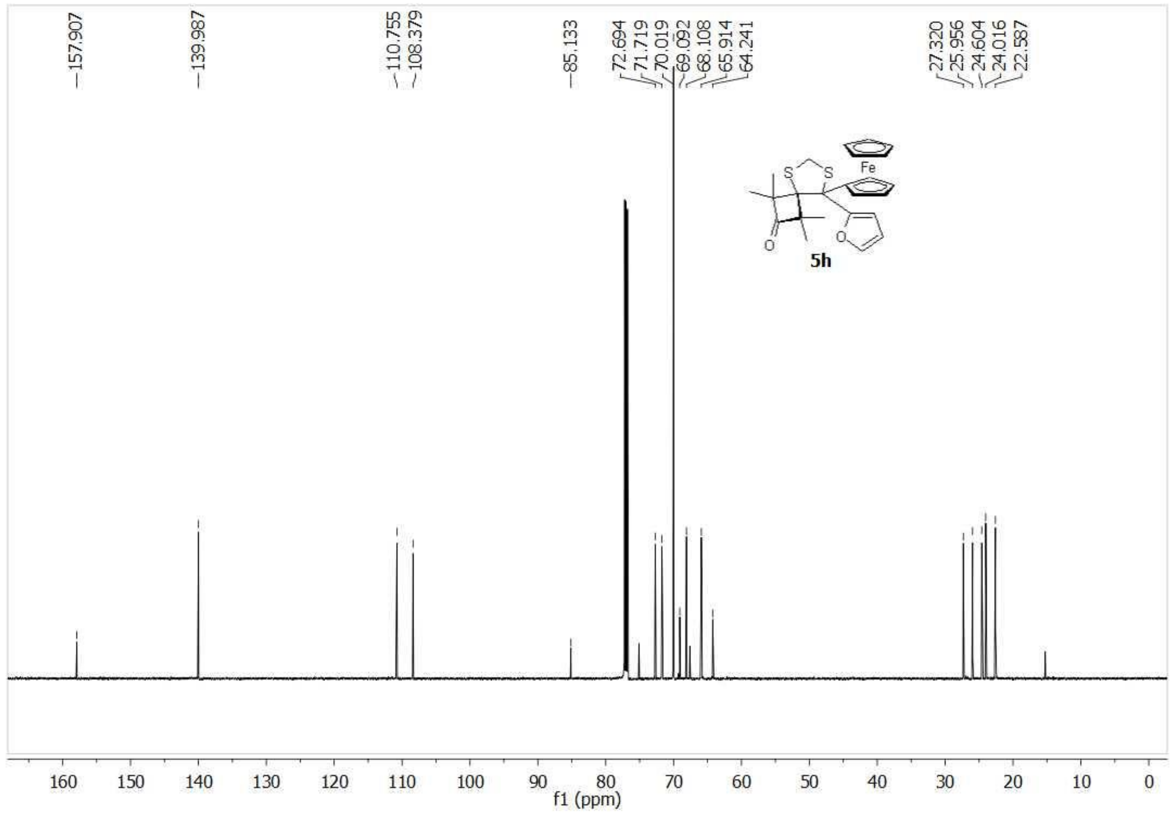

Figure S16. The ^{13}C NMR spectrum of compound **5h**.

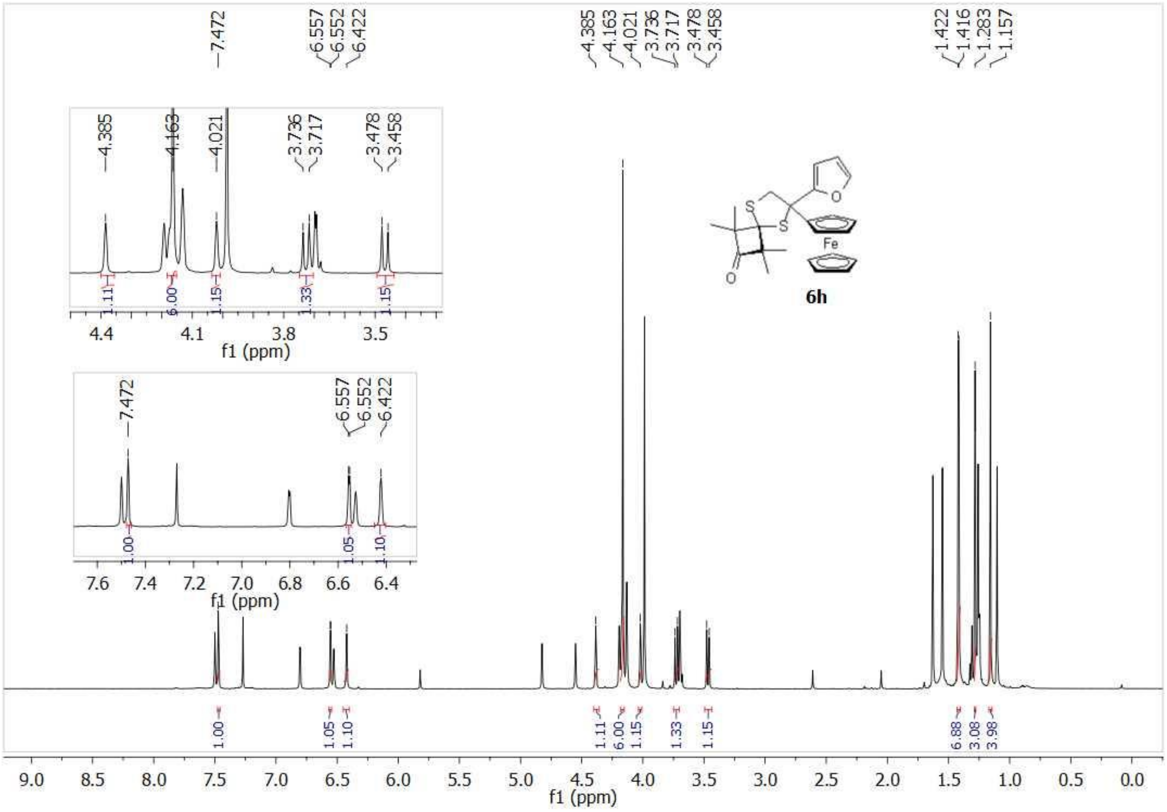

Figure S17. The ^{1}H NMR spectrum of compound **6h**.

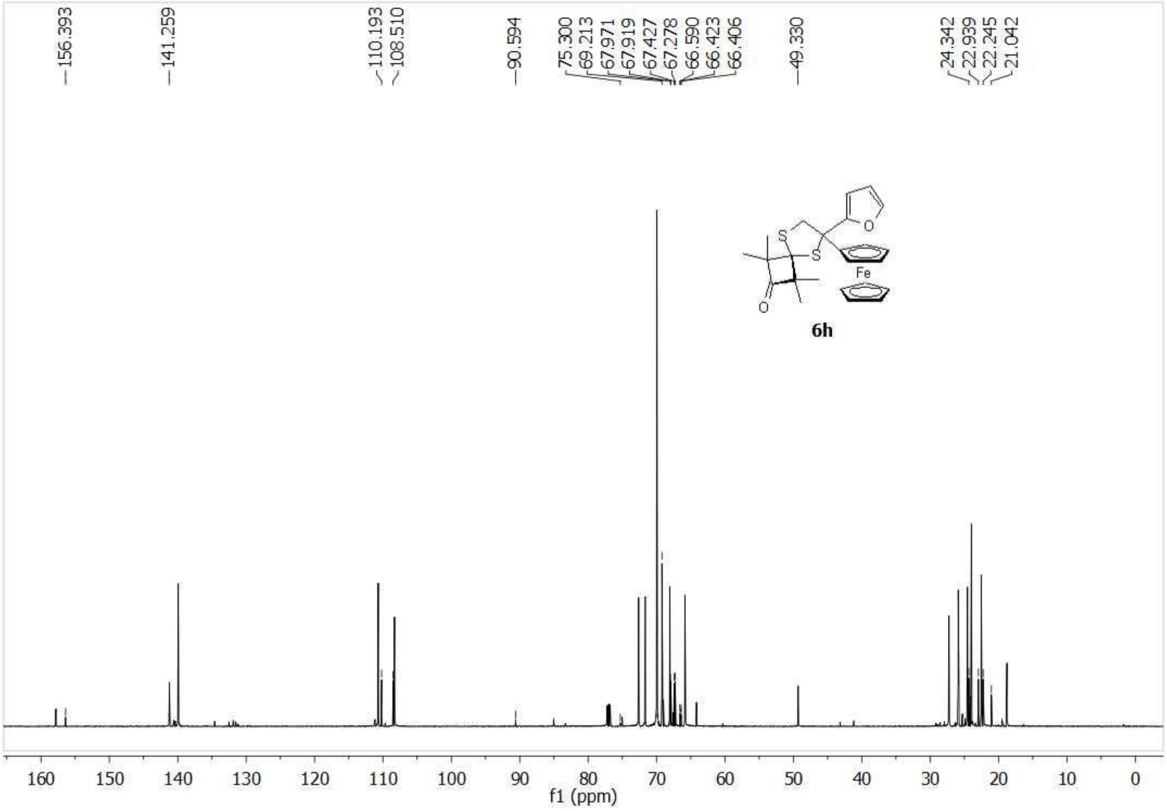

Figure S18. The ^{13}C NMR spectrum of compound **6h**.

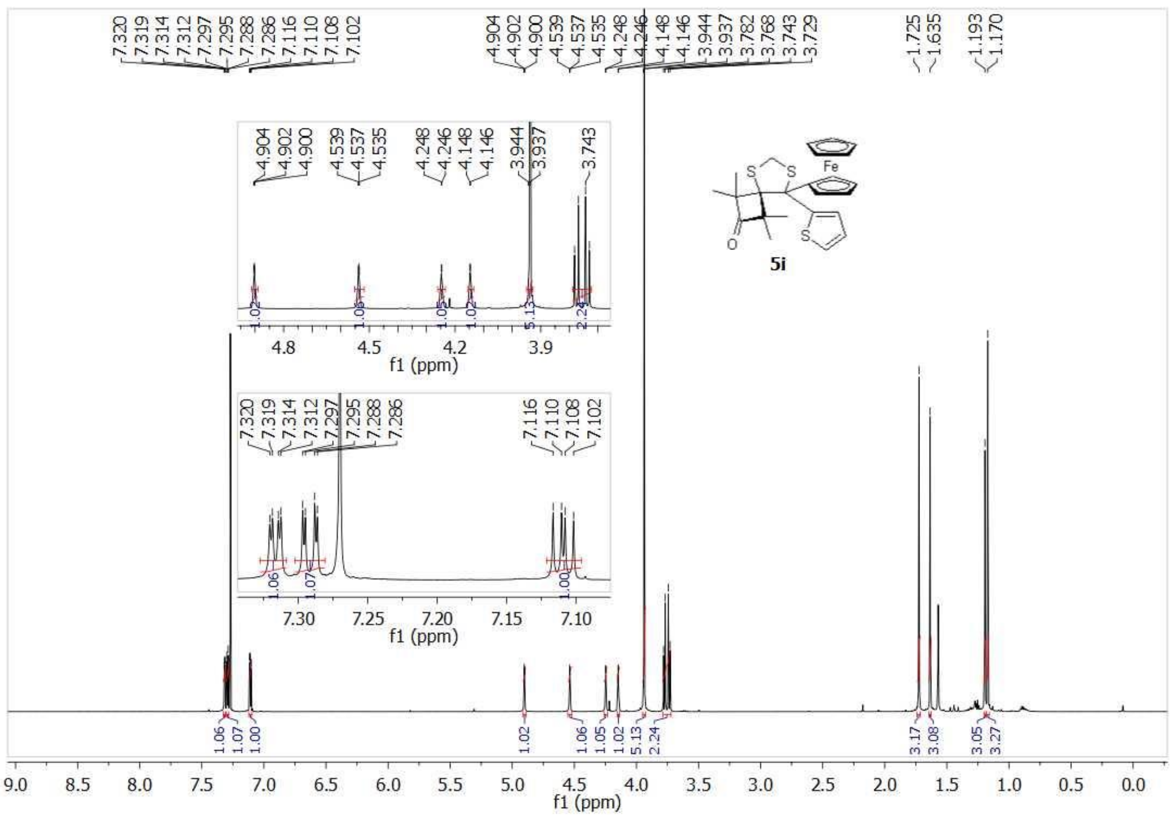

Figure S19. The ^{1}H NMR spectrum of compound **5i**.

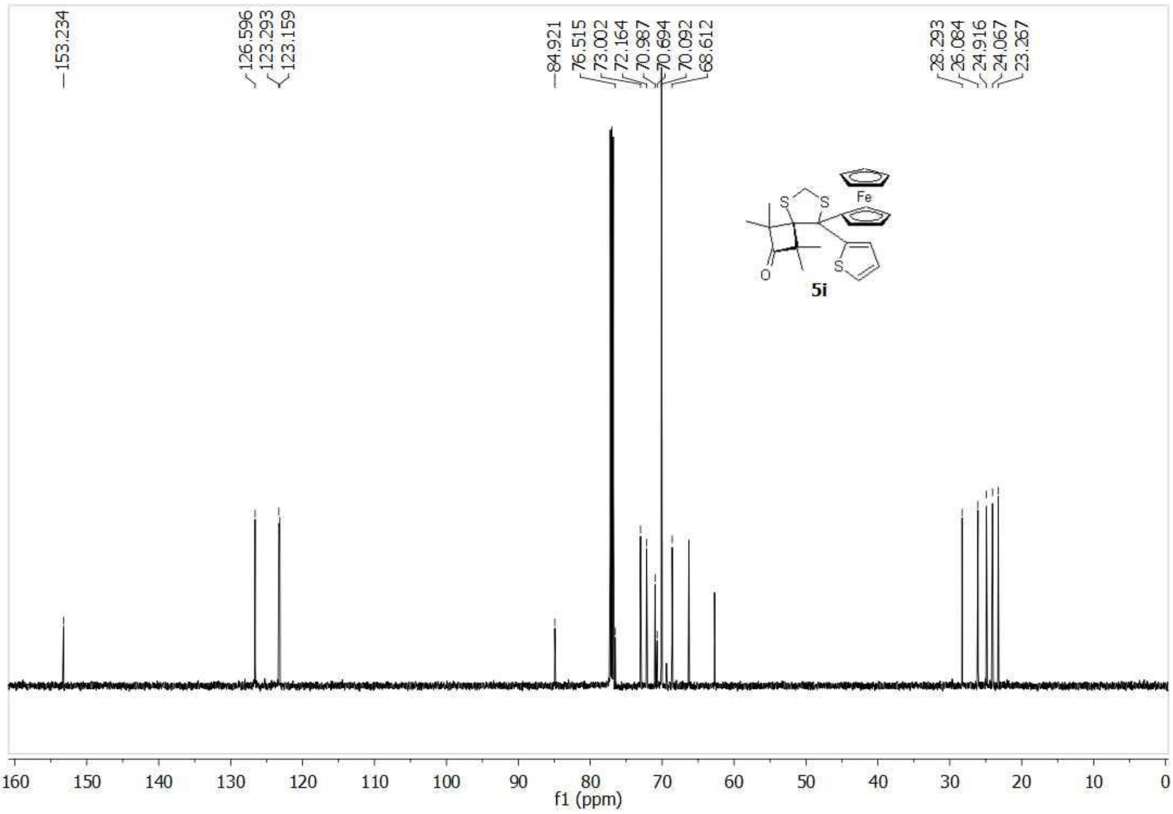

Figure S20. The ^{13}C NMR spectrum of compound **5i**.

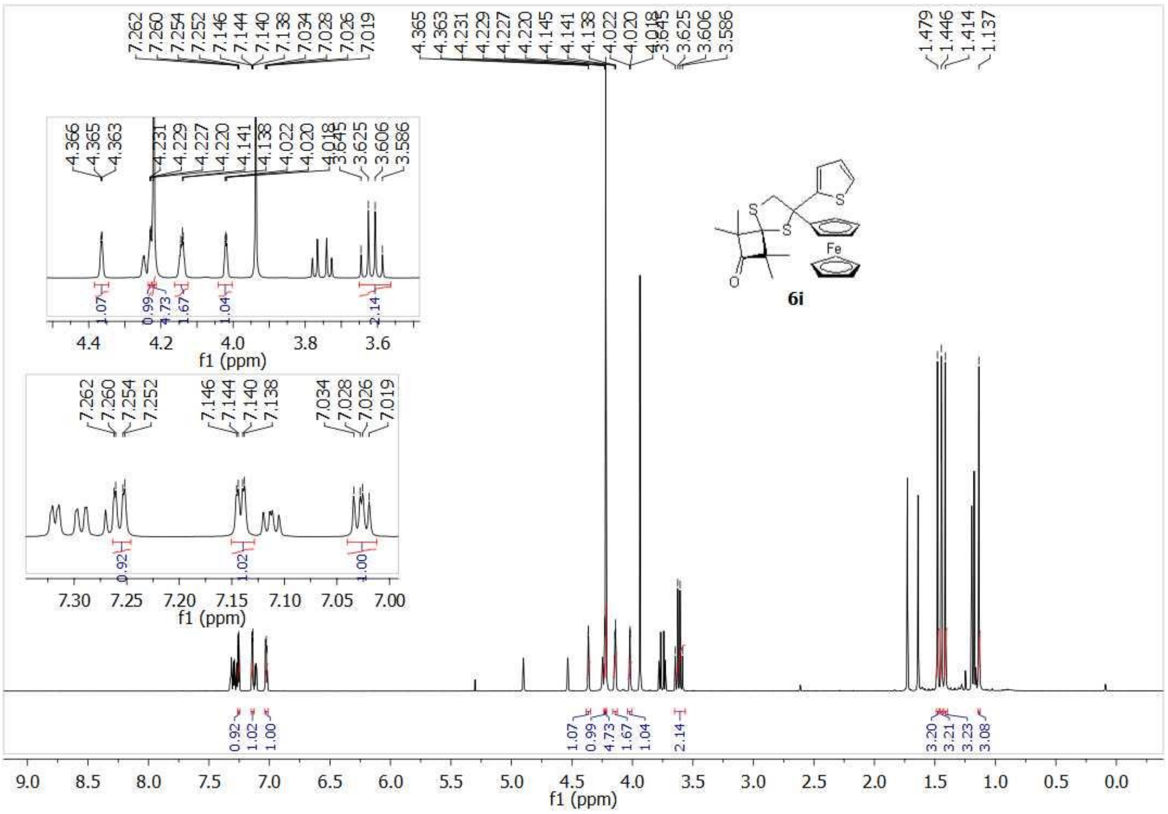

Figure S21. The ^{1}H NMR spectrum of compound **6i**.

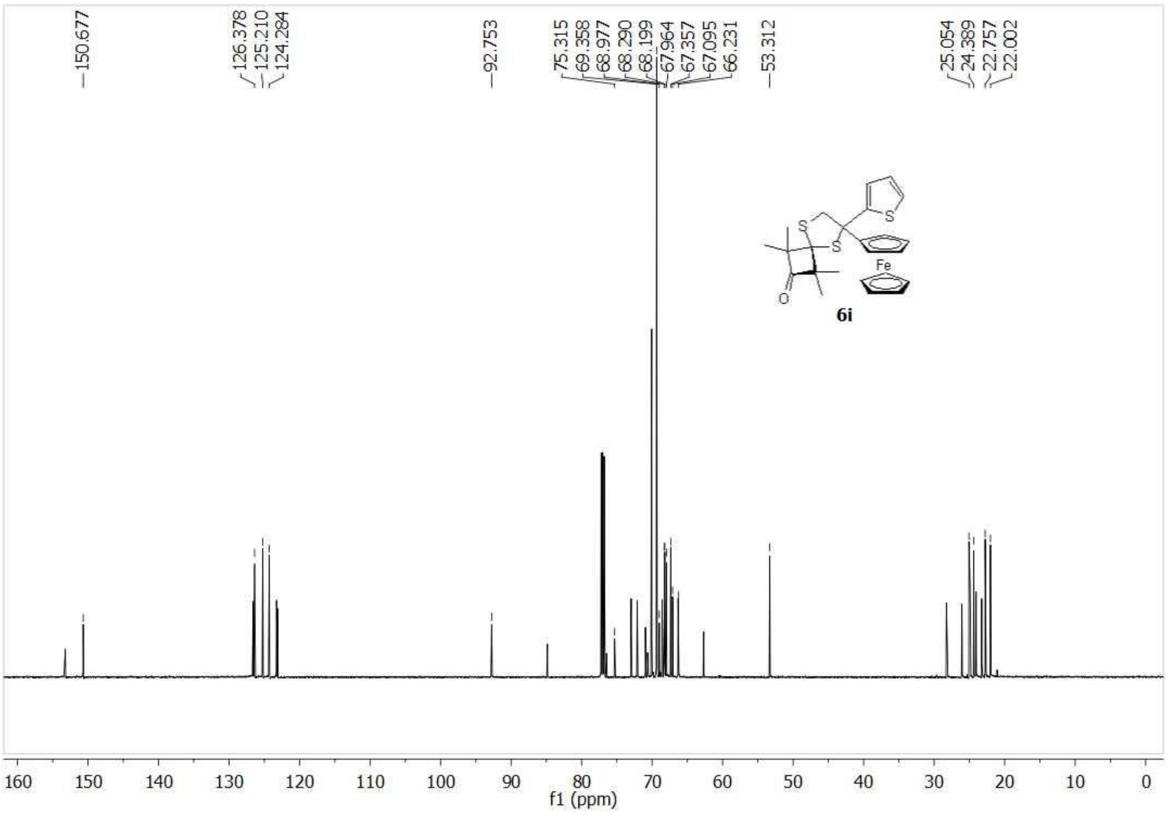

Figure S22. The ^{13}C NMR spectrum of compound **6i**.

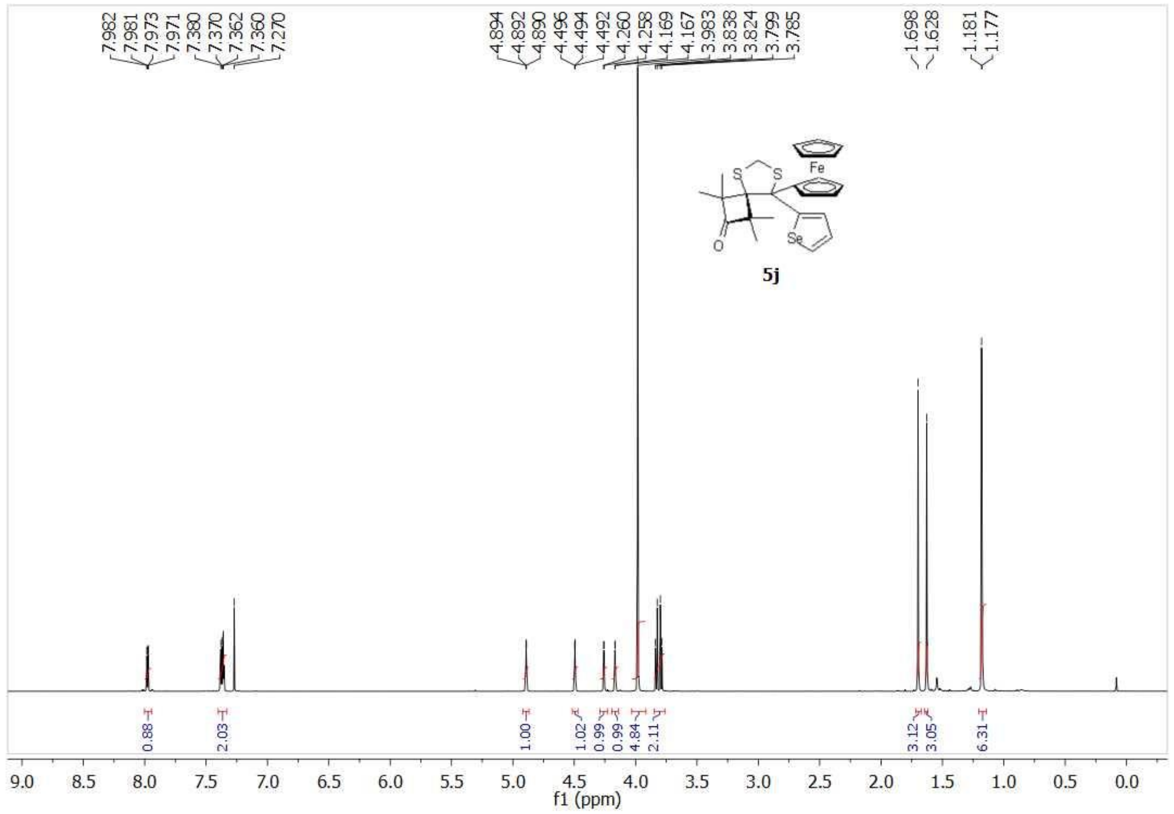

Figure S23. The ^{1}H NMR spectrum of compound **5j**.

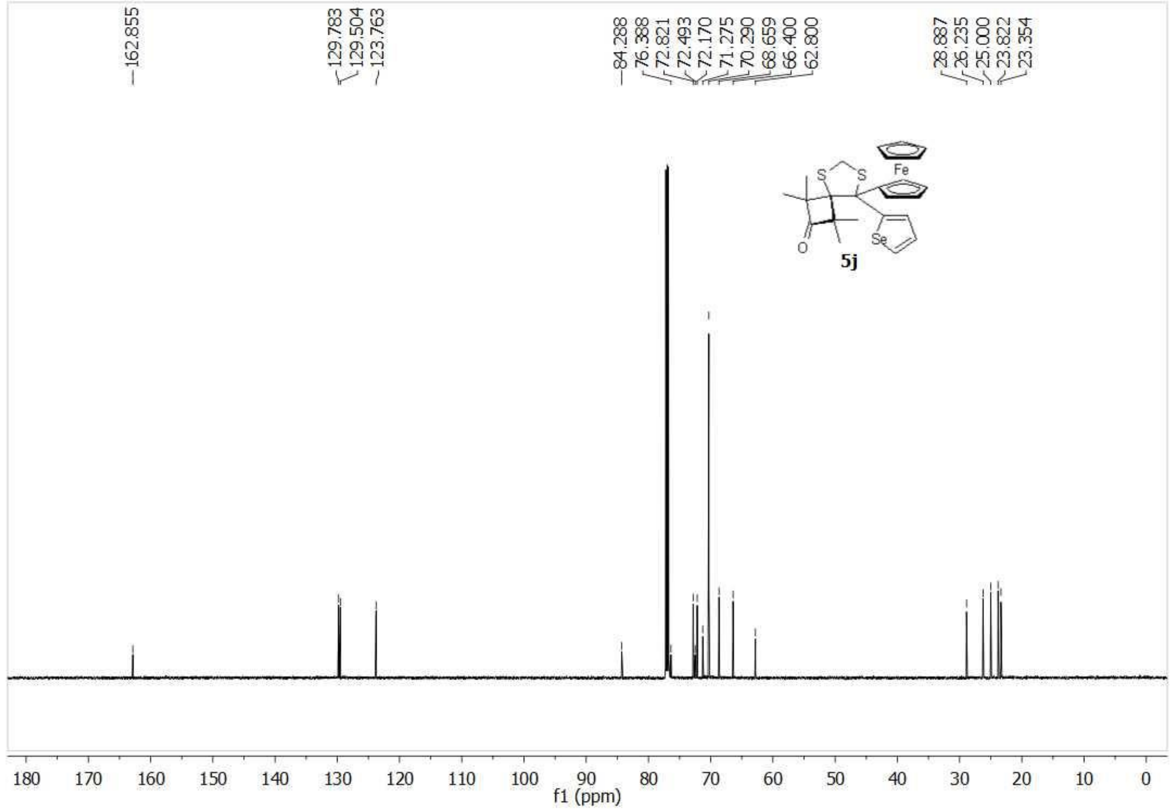

Figure S24. The ^{13}C NMR spectrum of compound **5j**.

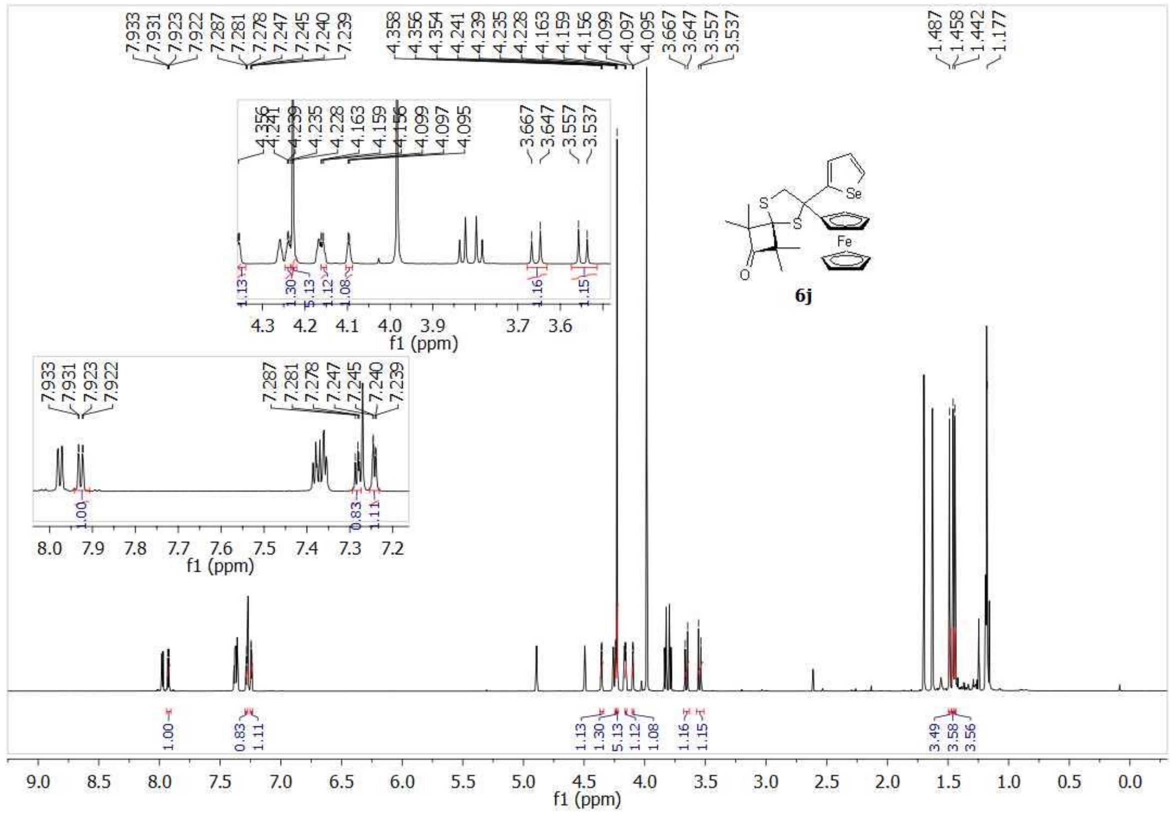

Figure S25. The ^{1}H NMR spectrum of compound **6j**.

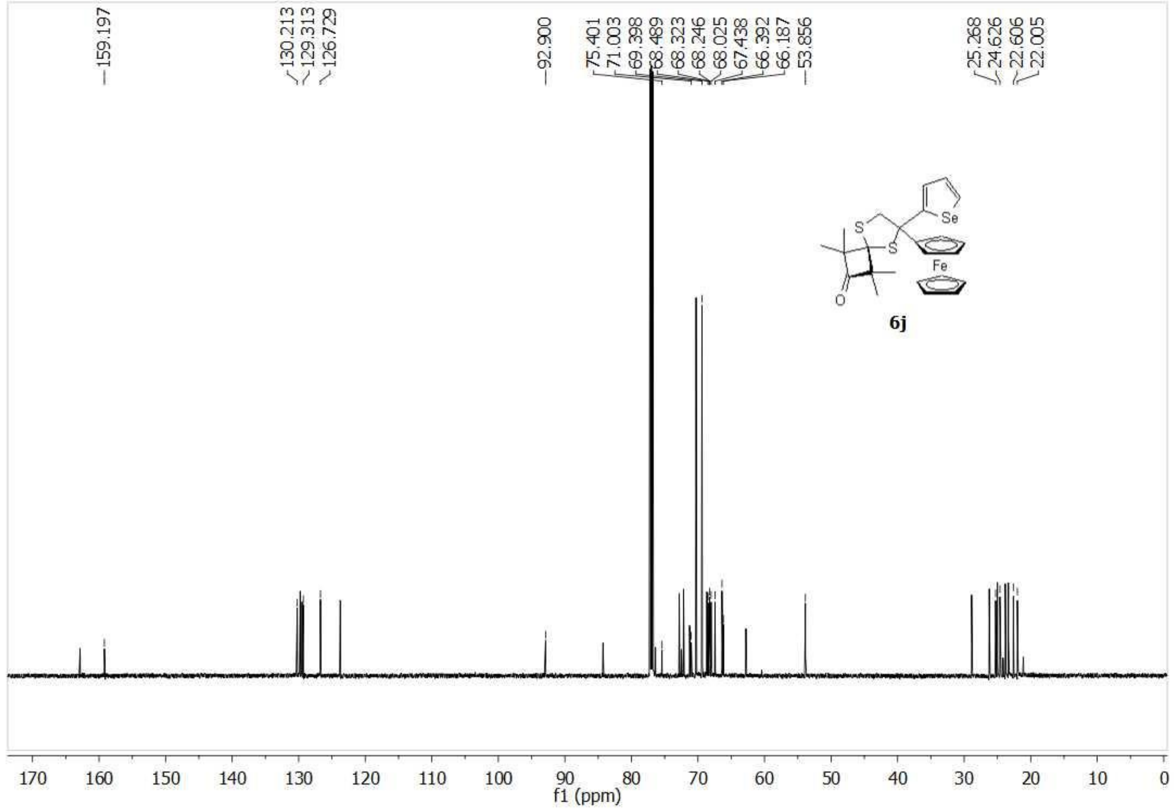

Figure S26. The ^{13}C NMR spectrum of compound **6j**.

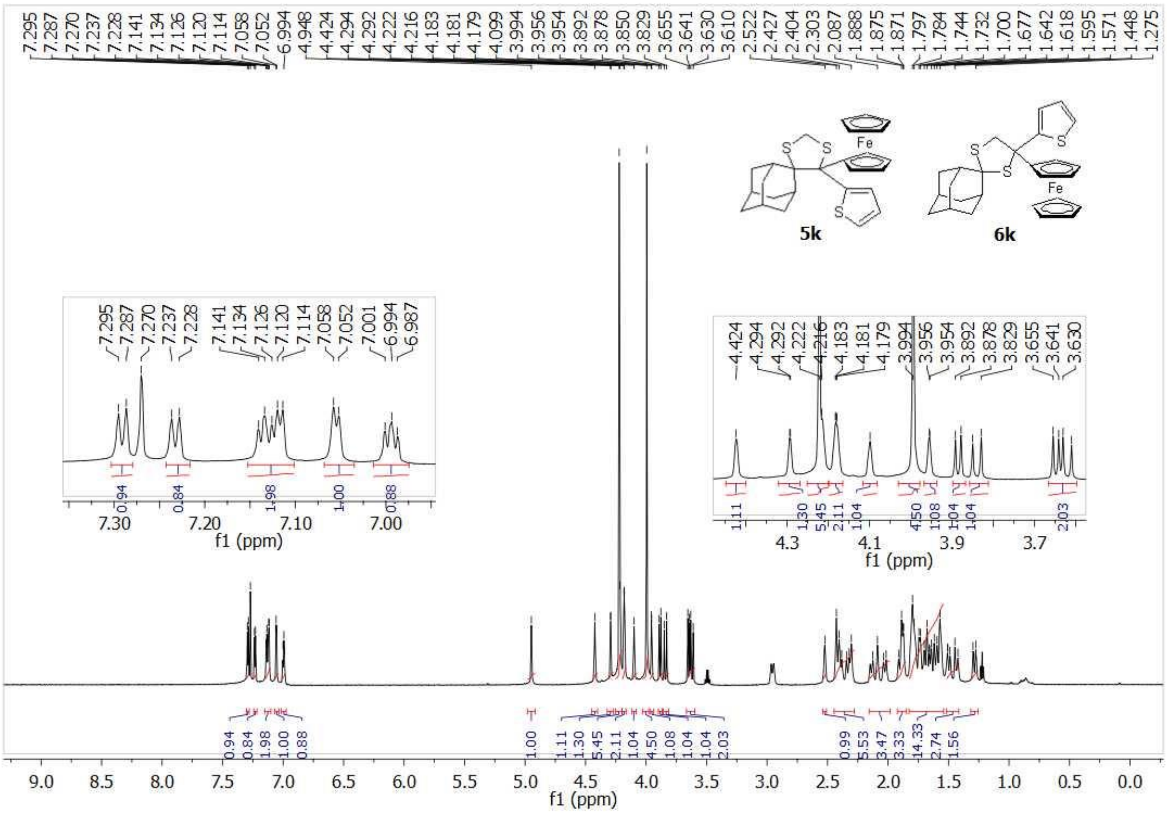

Figure S27. The ^{1}H NMR spectrum of compound **5k, 6k**.

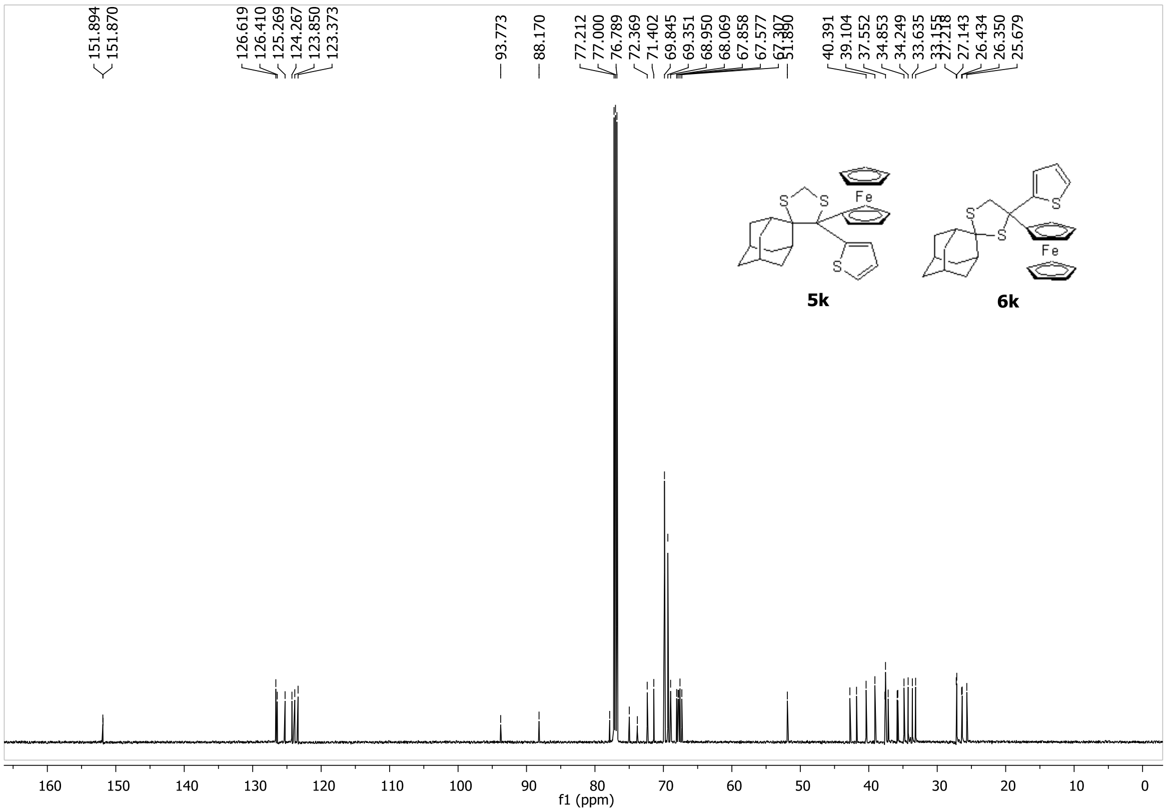

Figure S28. The ^{13}C NMR spectrum of compound **5k, 6k**.

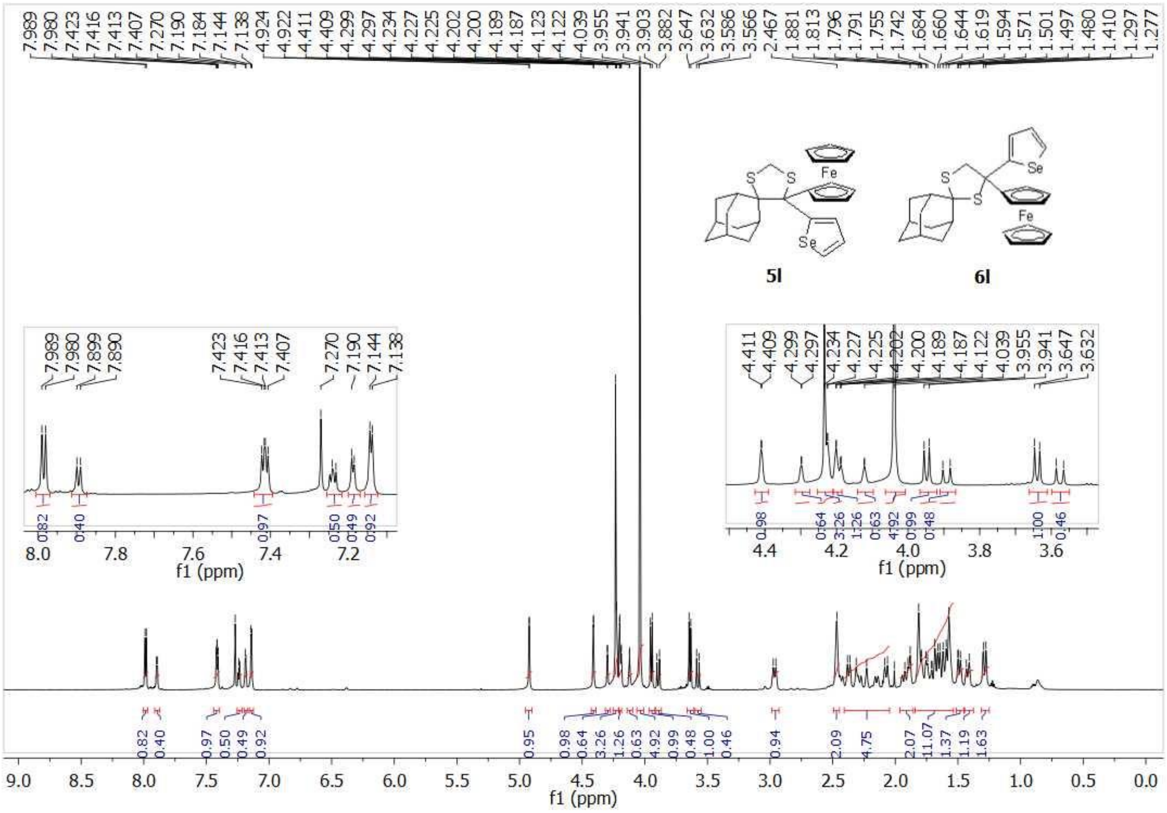

Figure S29. The ^{1}H NMR spectrum of compound **5l, 6l**.

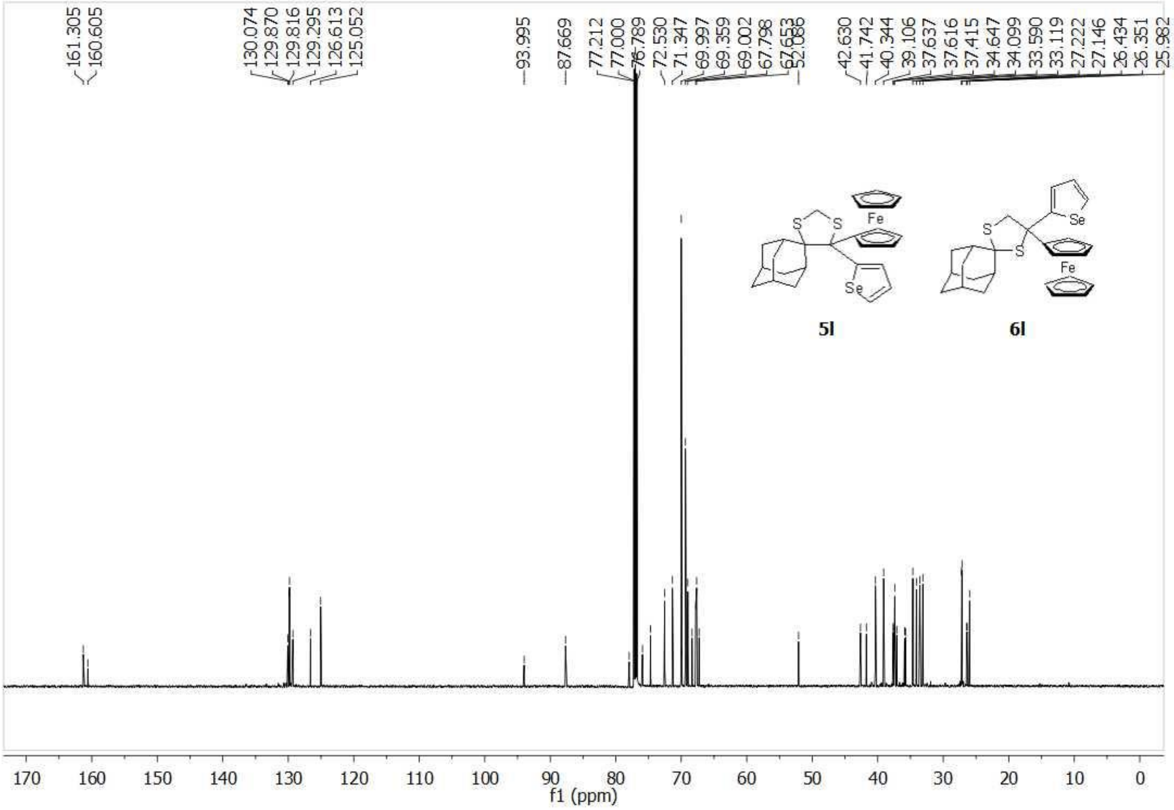

Figure S30. The ^{13}C NMR spectrum of compound **5l, 6l**.

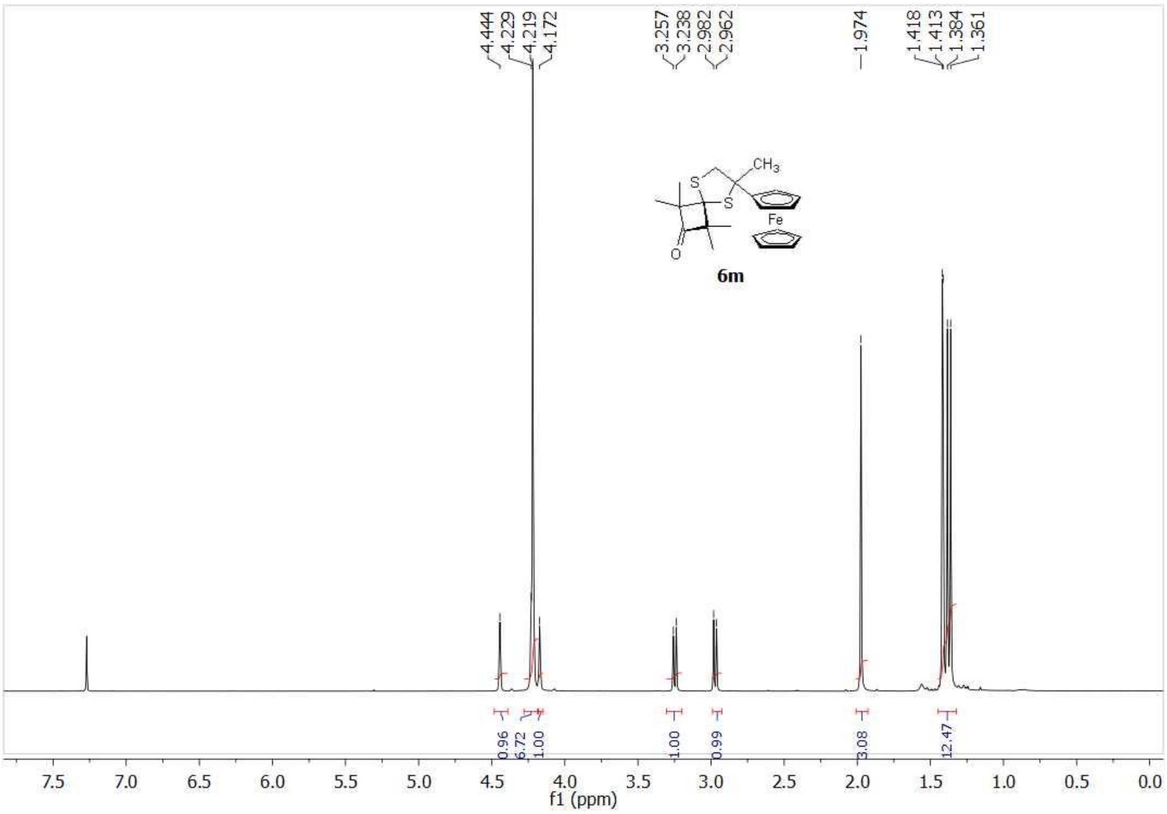

Figure S31. The ^{1}H NMR spectrum of compound **6m**.

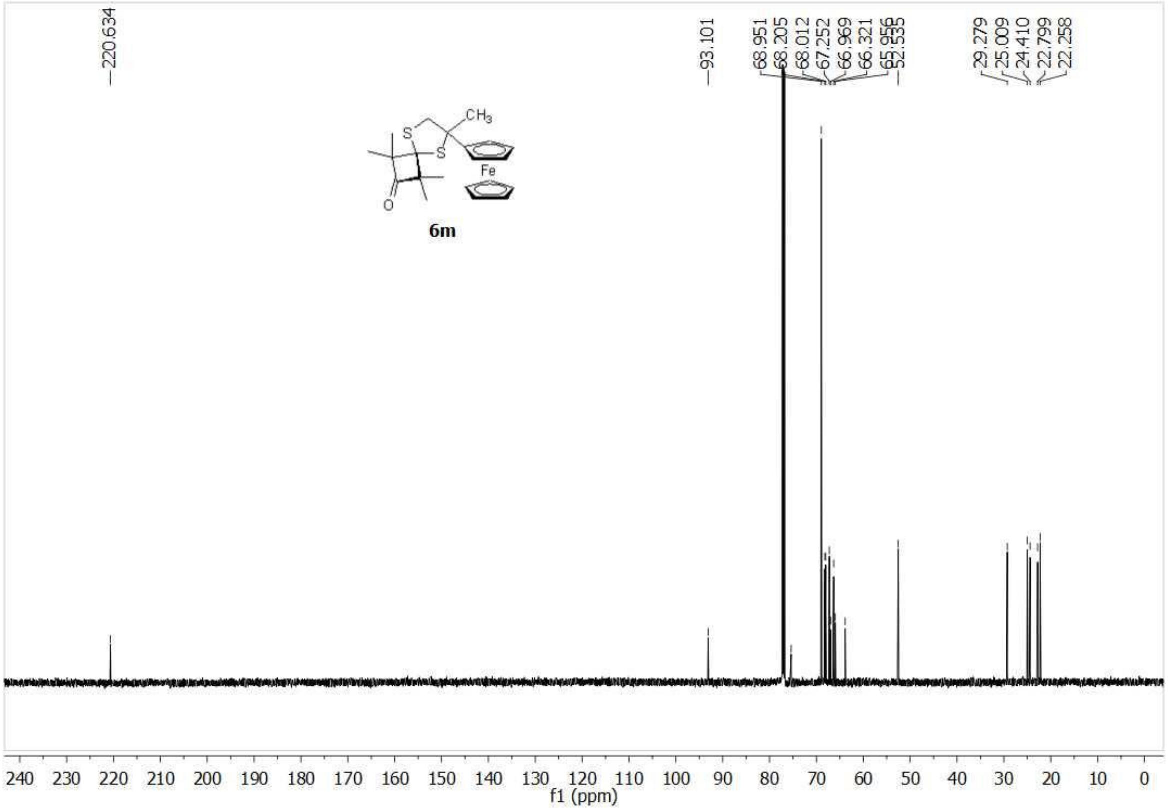

Figure S32. The ^{13}C NMR spectrum of compound **6m**.

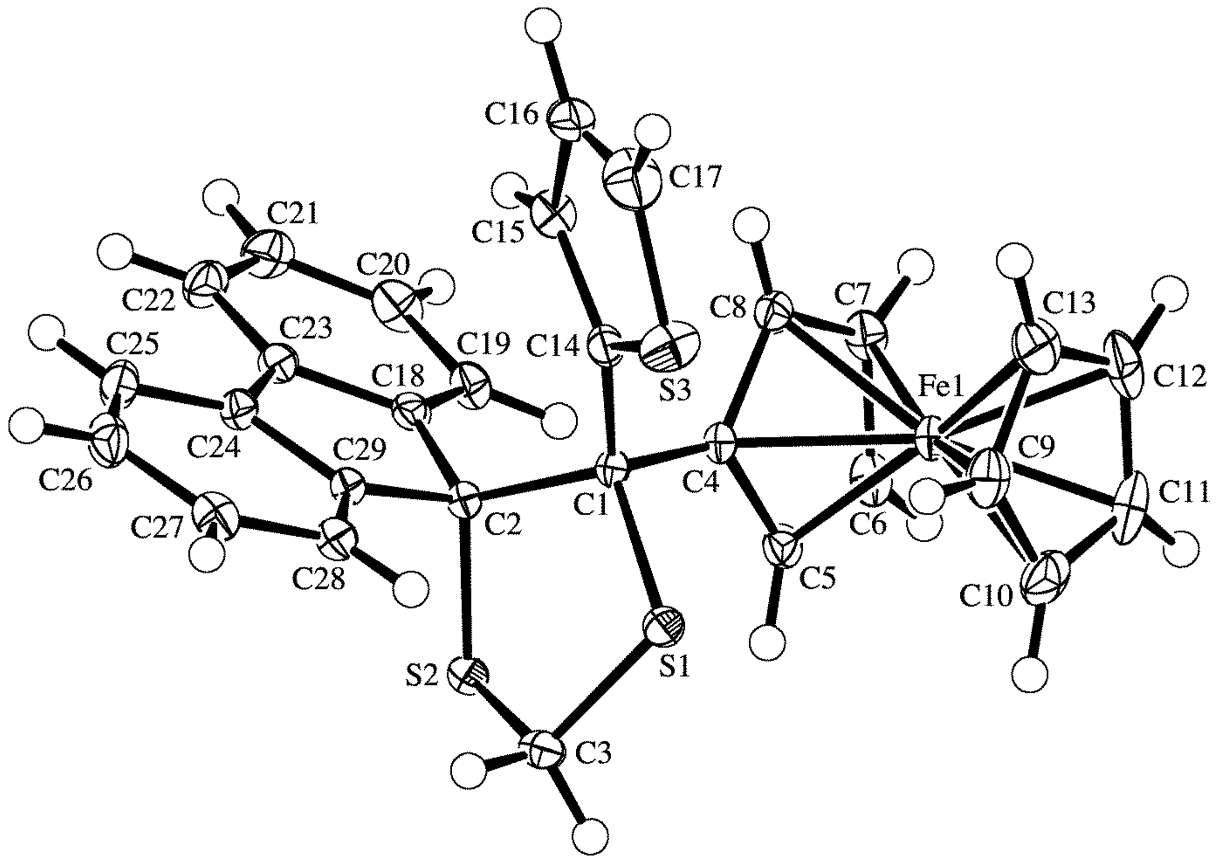

Figure S33. ORTEP Plot [S11] of the molecular structures of the ferrocenyl-substituted 1,3-dithiolane **5e** (with 50% probability ellipsoids; arbitrary numbering of the atoms; only the major disorder conformation of the thiophene ring is shown)

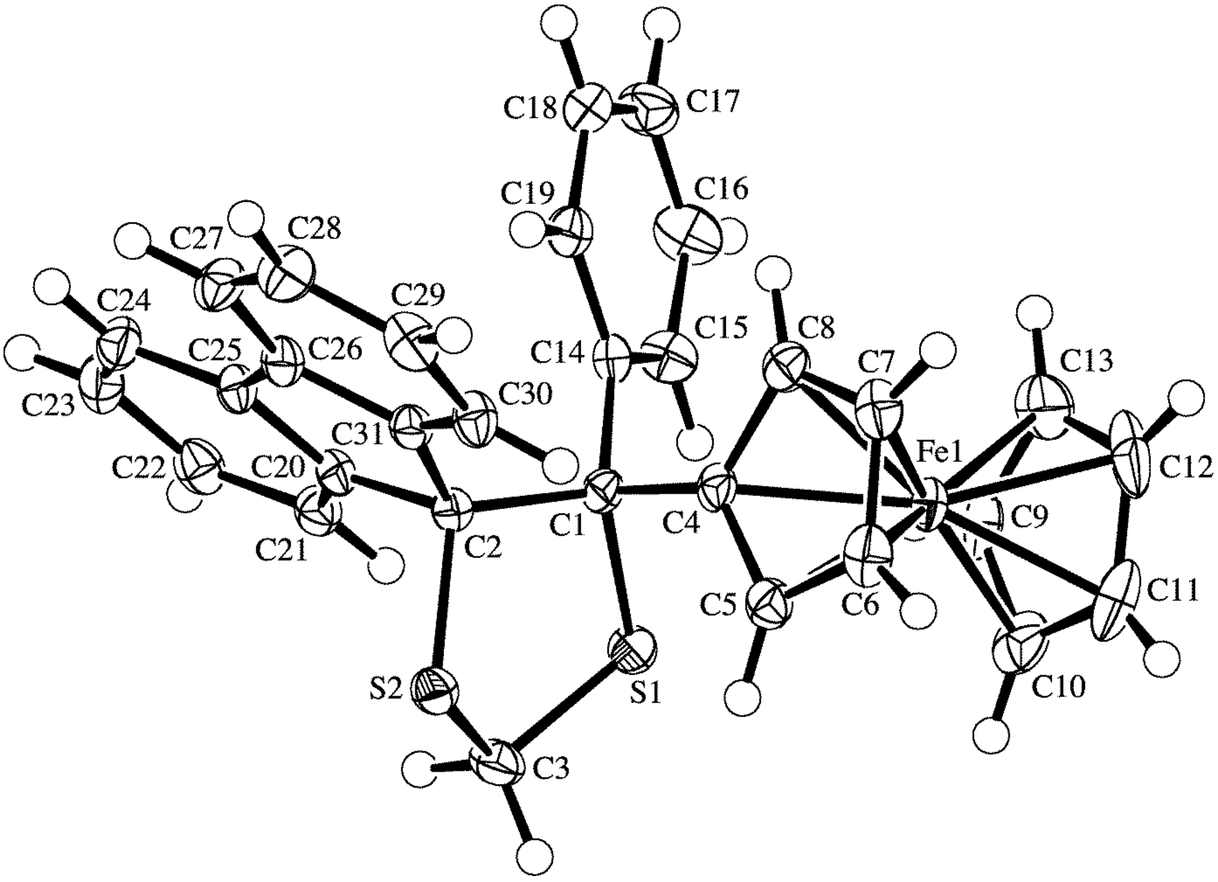

Figure S34. ORTEP Plot [S11] of the molecular structures of the ferrocenyl-substituted 1,3-dithiolane **5g** (with 50% probability ellipsoids; arbitrary numbering of the atoms)
